# Hepatic Fis1 regulates mitochondrial integrated stress response and improves metabolic homeostasis

**DOI:** 10.1172/jci.insight.150041

**Published:** 2022-02-22

**Authors:** Yae-Huei Liou, Jean Personnaz, David Jacobi, Nelson H. Knudsen, Mayer M. Chalom, Kyle A. Starost, Israel C. Nnah, Chih-Hao Lee

**Affiliations:** 1 Department of Molecular Metabolism, Division of Biological Sciences, Harvard T.H. Chan School of Public Health, Boston, Massachusetts, USA.; 2 Graduate School of Biomedical Sciences, Department of Cellular, Molecular, and Developmental Biology, Tufts University School of Medicine, Boston, Massachusetts, USA.

**Keywords:** Metabolism, Glucose metabolism, Mitochondria, Obesity

## Abstract

Mitophagy and mitochondrial integrated stress response (ISR) are 2 primary protective mechanisms to maintain functional mitochondria. Whether these 2 processes are coordinately regulated remains unclear. Here we show that mitochondrial fission 1 protein (Fis1), which is required for completion of mitophagy, serves as a signaling hub linking mitophagy and ISR. In mouse hepatocytes, high fat diet (HFD) feeding induces unresolved oxidative stress, defective mitophagy and enhanced type I interferon (IFN-I) response implicated in promoting metabolic inflammation. Adenoviral-mediated acute hepatic Fis1 overexpression is sufficient to reduce oxidative damage and improve glucose homeostasis in HFD-fed mice. RNA-Seq analysis reveals that Fis1 triggers a retrograde mitochondria-to-nucleus communication upregulating ISR genes encoding anti-oxidant defense, redox homeostasis, and proteostasis pathways. Fis1-mediated ISR also suppresses expression of IFN-I–stimulated genes through activating transcription factor 5 (Atf5), which inhibits the transactivation activity of interferon regulatory factor 3 (Irf3) known to control IFN-I production. Metabolite analysis demonstrates that Fis1 activation leads to accumulation of fumarate, a TCA cycle intermediate capable of increasing Atf5 activity. Consequently, hepatic *Atf5* overexpression or monomethyl fumarate (MMF) treatment improves glucose homeostasis in HFD-fed mice. Collectively, these results support the potential use of small molecules targeting the Fis1-Atf5 axis, such as MMF, to treat metabolic diseases.

## Graphical abstract

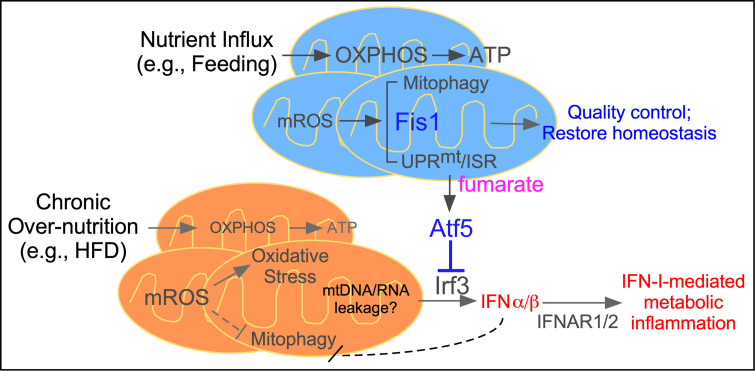

## Introduction

Mitochondrial oxidative phosphorylation (OXPHOS) is central to energy substrate utilization and ATP production. Dysregulated mitochondrial activity, notably increased oxidative stress, is often associated with obesity and metabolic diseases (1–3). Mitochondrial metabolism is also intimately linked to inflammatory reactions. This is exemplified by the metabolic reprogramming from oxidative to glycolytic metabolism during bacterial infection, balancing sufficient ATP production to fuel immune responses and the production of reactive oxygen species and synthesis of active metabolites to mediate effector functions of immune cells (4). In addition, mitochondria serve as a hub for the innate anti-viral immune response (5). The mitochondrial antiviral signaling protein (MAVS) located on the mitochondrial outer membrane integrates viral DNA/RNA-sensing signaling pathways, such as the retinoic acid-inducible gene-I (RIG-1) and cyclic GMP-AMP synthase/stimulator of interferon genes (cGAS/STING), and activates interferon regulatory factors 3 (IRF3) and 7 (IRF7) (5). Other viral sensing pathways, such as TLR3, also converge on IRF3/IRF7 to regulate the production of type I interferons (IFN-Is), including IFN-β and several subtypes of IFN-α, which induce the expression of IFN-stimulated genes (ISGs) in an autocrine or paracrine manner (6, 7). A prior study has demonstrated that increased IFN-I response induces CD8^+^ T cell-mediated metabolic inflammation in diet-induced obese mice to drive hepatic steatosis and insulin resistance (8). The triggers and cellular origins of IFN-I signaling have not been identified.

Mitochondrial dynamics, which include fusion, fission, and selective mitochondrial autophagy (mitophagy), is a key regulatory mechanism that maintains energy homeostasis and mitochondrial quality control (9, 10). Mitochondrial fusion creates an elongated network believed to increase metabolic efficiency, while fission leads to fragmentation of mitochondrial network that favors uncoupled respiration to reduce oxidative stress and marks damaged mitochondrial components to be removed by mitophagy. The physiological relevance of mitochondrial dynamics has been demonstrated in human diseases (11). Mutations in components of mitophagy, including *PINK* and *PARKIN*, are causative of Parkinson’s disease, whereas mutations in the mitochondrial fusion gene mitofusion 2 (*MFN2*) lead to peripheral neuropathy (12, 13). In mouse genetic models, liver-specific *Mfn2*^-/-^ mice have dysregulated ER function and insulin signaling (14). By contrast, hepatic *Mfn1*^-/-^ or *Dmn1l*^-/-^ (dynamin 1 like, Dmn1l or Drp1, a fission-promoting protein) mice are both protected from high fat diet (HFD)-induced insulin resistance (15, 16). These studies demonstrate the importance of the fusion-fission-mitophagy cycle in the maintenance of cellular function. In line with this, we have previously shown that in mouse liver, mitochondrial fusion and fission/mitophagy are linked to the fasted and fed states, respectively, and are subjected to circadian regulation (17). Liver-specific deletion of the master circadian clock regulator *Bmal1* causes enlarged mitochondria with elevated oxidative damage in hepatocytes and exacerbated hepatic lipid accumulation and insulin resistance in HFD-fed mice.

Mitochondrial unfolded protein response (UPR^mt^) is another principle homeostatic mechanism of mitochondrial function (18). The mitochondrial genome only encodes a small number of total mitochondrial proteins. The import of nuclear-encoded proteins is therefore a tightly regulated process. Chemicals that either inhibit mitochondrial protein translation or depolarize mitochondrial membrane potential (which blocks protein import) have been shown to trigger UPR^mt^. Initial work from *C*. *elegans* has indicated a mitochondrial-nuclear communication mechanism through activating transcription factor associated with stress-1 (ATFS-1), a transcription factor that translocates from the mitochondria to the nucleus to control the expression of protein chaperone and mitochondrial OXPHOS genes during UPR^mt^ (19, 20). There is, however, no clear ATFS-1 homologue in mammalian cells. Recent studies using comprehensive RNA-Seq, proteomic, and metabolomic analyses have suggested that mitochondrial stress inducers elicit an integrated stress response (ISR), which contains components of both UPR^mt^ and endoplasmic reticulum UPR (UPR^er^) controlled by shared upstream signaling, such as general control nonderepressible 2 (GCN2) kinase, and downstream effectors (21, 22). Pathways activated during ISR include amino acid biosynthesis, antioxidant mechanism/redox homeostasis, and 1-carbon metabolism/serine synthesis. These studies also implicate the involvement of stress responsive transcription factors, ATF4 and/or ATF5, in mediating the transcriptional events of ISR (21, 23). Thus, mitochondrial dynamics and ISR represent 2 protective mechanisms to maintain mitochondrial integrity. Whether and how these 2 pathways integrate in response to metabolic insults remain unclear.

The published work on ISR has been based on chemical stressors in immortalized cells, such as HeLa cells (21). The endogenous ISR-inducing signals and physiological relevance/importance of the ISR have not been addressed. In the current study, we show that enhanced Fis1 activity drives an ISR in hepatocytes. Although initially described as a mitochondrial fission factor, Fis1 is now known to be required for the completion of the mitophagy process (24, 25). Our results also suggest that Fis1-stimulated ISR suppresses IFN-I–mediated metabolic inflammation through the downstream effector Atf5. Activation of the Fis1-Atf5 axis of the ISR in the liver is sufficient to restore systemic glucose homeostasis in mice fed an HFD.

## Results

### Sustained oxidative stress is associated with defective mitophagy and elevated IFN-I signaling in overnutrition.

**Figure 1:**
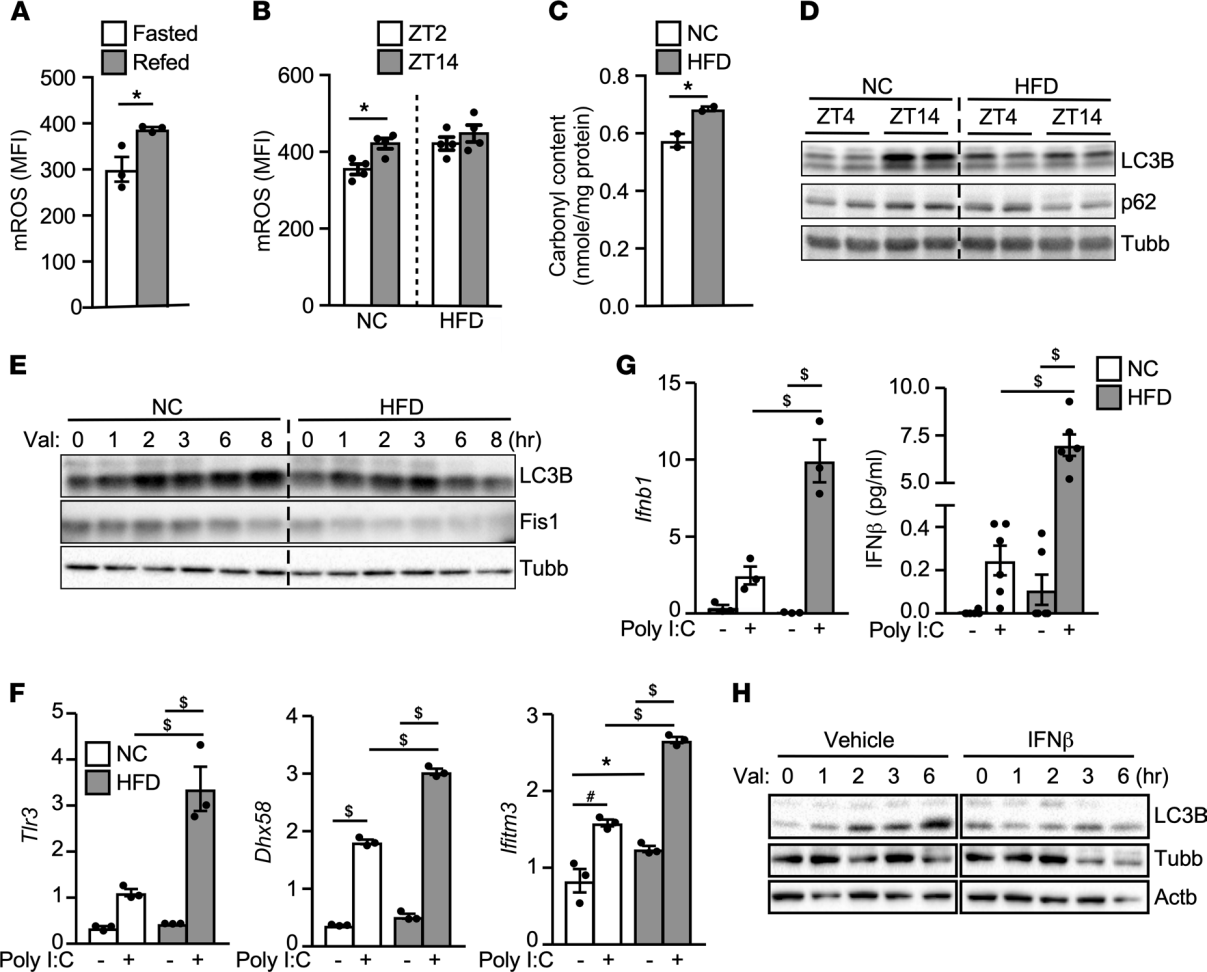
Overnutrition induces oxidative stress and impairs mitophagy in the liver. (**A**) Liver mROS production in fasted/refed mice. Mice were fasted overnight, followed by a 5-hour fasting or refeeding. Liver mitochondria were stained with MitoSOX Red plus 20 mM sodium succinate (to induce mROS production) for FACS analysis. MFI, mean fluorescence intensity from FACS analysis. *n* = 3, experiments repeated twice. (**B**) mROS levels in liver mitochondria isolated at diurnal fasting (Zeitgeber time 2, or ZT2 = 8 am) and feeding (ZT14 = 8 pm) periods. Mice were fed a NC or HFD for 8 weeks. *n* = 3–5, experiments repeated twice. (**C**) Protein carbonyl content in livers of NC- or HFD-fed mice at ZT2, repeated twice. (**D**) LC3B and p62 immunoblotting in NC or HFD livers at ZT4 and ZT14. *n* = 2, repeated twice. Beta tubulin (Tubb) included for loading control. (**E**) LC3B and Fis1 immunoblotting in primary hepatocytes from NC- or HFD-fed mice with 10 μM valinomycin at indicated time points, repeated 3 times. (**F**) Relative expression of IFN-I signaling genes by real-time PCR. Primary hepatocytes from NC- or HFD-fed mice were transfected with/without 100 ng/well Poly I:C for 16 hours. *n* = 3, repeated 3 times. (**G**) *Ifnb1* gene expression (*n* = 3) and IFN-β protein secretion (*n* = 6, normalized to cellular protein content) from the condition described in **F**. (**H**) LC3B immunoblotting in primary hepatocytes stimulated with 10 ng/mL IFN-β for 16 hours, followed by 10 μM valinomycin time course treatment. Tubb and beta actin (Actb): loading controls. Experiments repeated twice. Values are mean ± SEM. Significance of **A**–**C** were determined by unpaired, 2-tailed Student’s *t* test; and of **F** and **G** by 1-way ANOVA followed by Holm-Šidák multiple comparisons test. *P < 0.05; ^#^*P* < 0.01; ^$^*P <* 0.001.

A byproduct of mitochondrial oxidative metabolism is the generation of mitochondrial reactive oxygen species (mROS) (2). In mice on a normal chow (NC), the level of mROS in the liver was increased by refeeding (Figure 1A) or during the diurnal feeding cycle (zeitgeber time 14 [ZT14] or 8 pm), compared with the fasting cycle (ZT2 or 8 am; Figure 1B), in line with the notion that increased nutrient influx drives mROS production. Livers of mice on an HFD had similarly elevated mROS levels at both ZT4 and ZT14 and showed increased oxidative protein modification compared with livers of chow-fed mice, determined by protein carbonylation (Figure 1C).

We have previously shown that circadian regulation of mitophagy genes at the feeding cycle plays an important role in managing oxidative stress in the liver (17). Deletion of the master circadian regulator *Bmal1* gene in the liver led to excessive oxidative damage and swollen mitochondria (17). Enlarged mitochondrial morphology was also evident in livers from HFD mice (Supplemental Figure 1A; supplemental material available online with this article; https://doi.org/10.1172/jci.insight.150041DS1). Western blotting revealed that the levels of the mitophagy/autophagy markers, LC3B and p62, were higher during feeding compared with fasting in the liver of chow-fed mice (comparing ZT14 to ZT4; Figure 1D). This dynamic change in LC3B/p62 was lost in the liver of HFD-fed mice. To assess whether mitophagy was suppressed by overnutrition, primary hepatocytes were isolated from NC- and HFD-fed mice and treated with valinomycin to induce mitophagy. LC3B protein was induced rapidly by valinomycin treatment in hepatocytes of NC-fed mice (Figure 1E). This effect was blunted by HFD feeding, which was associated with a decline in the Fis1 protein level, indicating a defect in mitophagy.

IFN-I signaling has been shown to increase in the liver of obese mice (8). It has also been implicated in promoting oxidative stress to inhibit autophagosome function and clearance of damaged mitochondria in monocytes, resulting in leakage of mitochondrial DNA and/or RNA that further activates the IFN-I response (26). We were able to confirm upregulation of ISGs in the liver and hepatocytes of HFD-fed mice, compared with NC-fed mice (Supplemental Figure 1, B and C). In addition, polyinosinic/polycytidylic acid (poly I:C), a TLR3 agonist, induced a stronger IFN-I response in hepatocytes from HFD-fed mice, assessed by the expression of viral RNA sensing molecules *Tlr3* and DExH-box helicase 58 (*Dhx58*) and the ISG IFN-induced transmembrane protein 3 (*Ifitm3*) (Figure 1F). The expression of *Ifnb1* and its protein product IFN-β was also significantly higher in hepatocytes of HFD-fed mice treated with poly I:C (Figure 1G), suggesting that hepatocytes are both targets and potential sources of IFN-Is in overnutrition. Lastly, IFN-β treatment blocked valinomycin-induced mitophagy in hepatocytes (Figure 1H). Thus, chronic overnutrition leads to sustained oxidative stress that is linked to defective mitophagy and enhanced IFN-I–mediated metabolic inflammation in the liver.

### Fis1 lowers mROS and restores metabolic homeostasis.

Fis1 has been shown to act in sequence with fission factors to couple oxidative stress-induced fission with the downstream mitophagy process (24). The Fis1 protein was downregulated during valinomycin treatment in hepatocytes from HFD-fed mice (Figure 1E), which could contribute to the defective mitophagy. Adenoviral-mediated shRNA knockdown of *Fis1* (Ad-shFis1) in hepatocytes increased mROS production and inhibited paraquat-induced mitophagy determined by accumulation of LC3B and p62 proteins, compared with control scrambled shRNA (Ad-shCtl; Supplemental Figure 2, A–C). In addition, reduced Fis1 activity by Ad-shFis1 in the liver of mice after a 4-week HFD feeding reduced glucose tolerance (Supplemental Figure 2, D and E, and Supplemental Table 1), indicating that defective mitophagy/increased oxidative stress may cause metabolic dysfunction during the course of chronic overnutrition.

**Figure 2:**
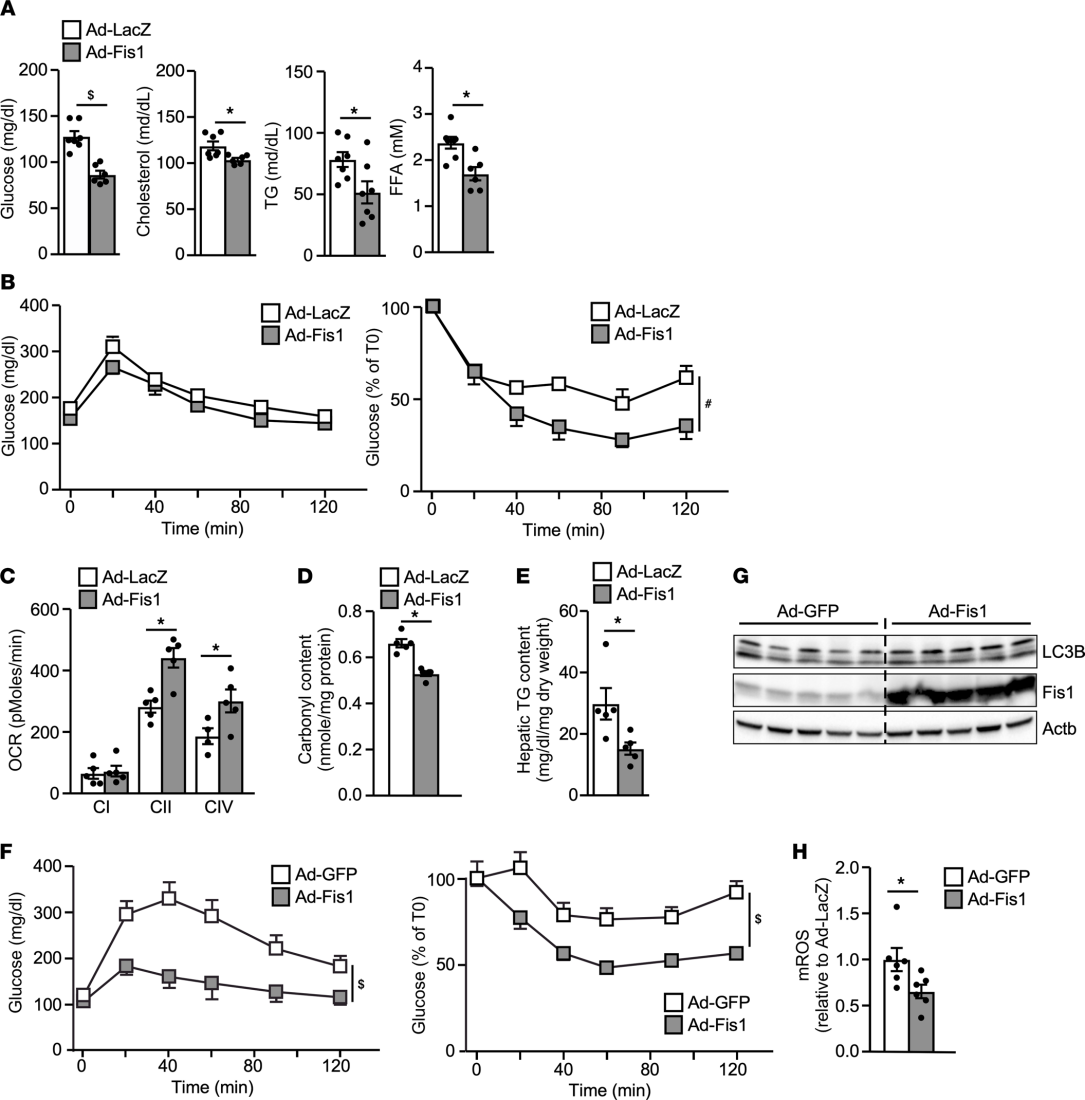
Hepatic Fis1 overexpression improves glucose homeostasis. (**A**) Levels of fasting glucose and serum lipids of Ad-LacZ– (control) or Ad-Fis1–infected male mice on a HFD for 4 weeks. Mice were fasted for 4 hours. *n* = 6–7, repeated in 2 cohorts. (**B**) GTT (left) and ITT (right) of Ad-LacZ or Ad-Fis1 infected male mice fed a HFD for 4 weeks. *n* = 7. (**C**) Electron flow assay using Seahorse bioanalyzer with mitochondria isolated from livers of Ad-LacZ– and Ad-Fis1–infected HFD-fed mice. Complex I (C-I) respiration was measured using pyruvate and malate as substrates and blocked with rotenone. Complex II (C-II) was measured using succinate as substrate and blocked with antimycin A. Complex IV (C-IV) respiration was measured by injecting tetramethyl-p-phenylenediamine/ascorbate. *n* = 5. OCR, oxygen consumption rate. (**D**) Protein carbonylation in livers of Ad-Lacz– or Ad-Fis1–infected mice. *n* = 4-5. (**E**) Liver TG content of Ad-LacZ– and Ad-Fis1–infected mice. *n* = 6-7. (**F**) GTT and ITT of Ad-GFP– (control) or Ad-Fis1–infected HFD-fed male mice (12 weeks HFD). *n* = 6 for 1 cohort. (**G**) Immunoblotting showing LC3B and Fis1 protein levels. HFD-fed male mice (12 weeks HFD) were infected with Ad-LacZ or Ad-Fis1. Liver samples were collected 7 days after infection. Actb protein level served as a loading control. *n* = 5. (**H**) Relative mROS production determined by MitoSOX Red. Mitochondria were isolated from livers of mice 7 days after infection. Ad-GFP was set as 1. *n* = 6. Values are presented as mean ± SEM. Significance was determined by 2-way ANOVA for GTT and ITT and unpaired, 2-tailed Student’s *t* test for 2 group comparisons. *P < 0.05; ^#^*P* < 0.01; ^$^*P* < 0.001.

To determine whether an acute increase in Fis1 activity in the liver could restore metabolic homeostasis, adenoviral *Fis1*-mediated hepatic overexpression (Ad-Fis1, Ad-LacZ, or Ad-GFP as a control) was employed. Enhanced Fis1 activity in the liver by Ad-Fis1 led to a reduction in the level of fasting glucose and serum lipids, including cholesterol, triglycerides (TGs) and free fatty acids (FFAs) in mice on an HFD for 4 weeks (Figure 2A, and Supplemental Table 2). Ad-Fis1–transduced mice also showed an improvement in the insulin tolerance test (ITT; Figure 2B), while there was no difference in the glucose tolerance test (GTT; Figure 2B). Consistent with the effect of mitophagy on mitochondrial quality control, mitochondrial respiration was enhanced in the Ad-Fis1 liver when given complexes II and IV substrates, succinate, and ascorbate/tetramethyl-*p*-phenylenediamine, respectively (Figure 2C). Consequently, hepatic oxidative damage (protein carbonylation) and TG content were reduced by Ad-Fis1 (Figure 2, D and E). In mice fed an HFD for 12 weeks to induce more profound obesity and metabolic dysregulation, Ad-Fis1 significantly increased both glucose and insulin tolerance compared with control mice (Figure 2F). As expected, Ad-Fis1 increased hepatic LC3B levels and suppressed mROS production (Figure 2, G and H). These results suggest that enhanced hepatic Fis1 activity in diet-induced obese mice restores mitochondrial function and improves lipid and glucose homeostasis.

### Fis1 overexpression triggers ISR.

To gain insight into mechanisms through which Fis1 regulates hepatic metabolism, gene expression profiling was determined by RNA-Seq analyses in hepatocytes infected with Ad-Fis1, compared with the Ad-LacZ control (Supplemental Figure 3A). Ad-Dmn1l was also included for comparison, as mitochondrial fission preceded mitophagy. In a low nutrient medium, Ad-LacZ-infected control hepatocytes exhibited an elongated mitochondrial network, while Ad-Fis1– and Ad-Dmn1l–infected hepatocytes maintained a fragmented morphology (Supplemental Figure 3B). For data quality control, results of hepatocytes infected with the same titer of Ad-LacZ from different virus preparations in 2 separate RNA-Seq experiments were compared, which showed a similar expression profile (Supplemental Figure 3C). *Fis1* overexpression led to substantial changes in gene expression (2620 genes, FDR < 0.001), while *Dmn1l* overexpression only affected a small number of genes (Supplemental Figure 3D, and Supplemental Data Set 1), indicating a specific role for Fis1-mediated mitophagy in inducing a retrograde mitochondrial-nuclear communication.

**Figure 3:**
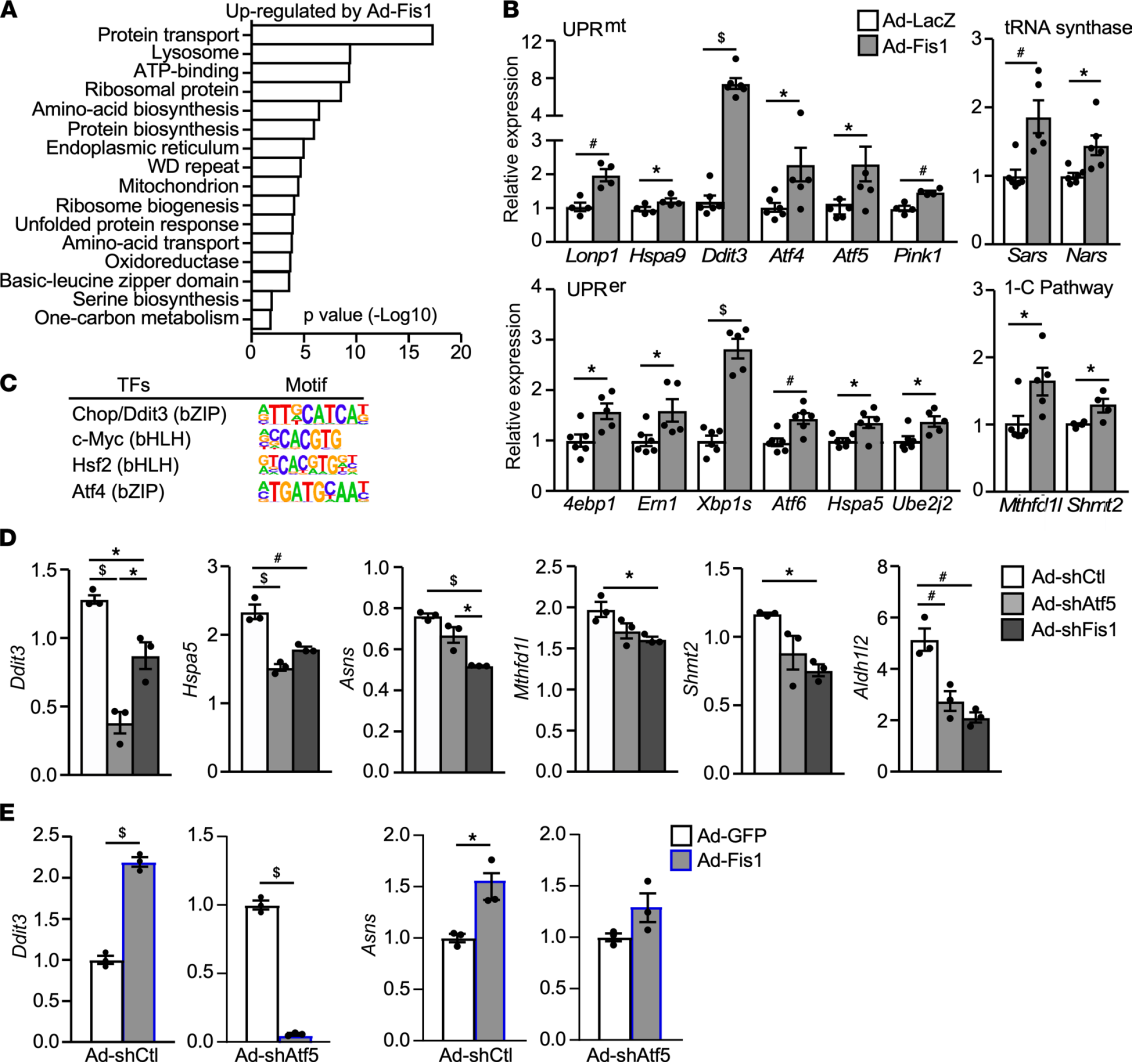
Fis1 overexpression induces an ISR. (**A**) Functional clustering analysis of 1236 genes upregulated (FDR *P* < 0.001) by Ad-Fis1 versus Ad-LacZ in primary hepatocytes identified by RNA-Seq. *n* = 4 in 1 experiment. (**B**) Validation of RNA-Seq results for ISR genes in UPR^mt^, UPR^er^, tRNA synthesis, and 1-carbon metabolism (1-C) pathway in primary hepatocytes infected with Ad-Fis1 or Ad-LacZ by real-time qPCR. *n* = 4–6. (**C**) HOMER motif analysis to identify potential transcription factor binding sites on promoters of Ad-Fis1–upregulated genes. (**D**) Assessing the effects of knocking down *Atf5* or *Fis1* on ISR gene expression in hepatocytes by real-time PCR analysis. Primary hepatocytes were infected with Ad-shCtl, Ad-shAtf5, or Ad-shFis1 and cells were harvested 48 hours after infection. *36b4* was used for normalization to determine the relative expression. *n* = 3, repeated 4 times. (**E**) Assessing Atf5 as a Fis1 downstream effector in regulating hepatic ISR gene expression using real-time PCR. Primary hepatocytes were infected with Ad-shCtl or Ad-shAtf5; 8 hours later, cells were washed and infected with Ad-GFP or Ad-Fis1. Hepatocytes were cultured for an additional 40 hours. *n* = 3, repeated twice. Values are presented as mean ± SEM. Significance of **B** and **E** were determined by unpaired, 2-tailed Student’s *t* test; and of **D** by 1-way ANOVA followed by Holm-Šidák multiple comparisons test. *P < 0.05; ^#^*P* < 0.01; ^$^*P* < 0.001.

Gene ontology analysis of upregulated genes by Ad-Fis1 versus Ad-LacZ revealed that top enriched pathways included tRNA/amino-acid synthesis (e.g., *Sars* and *Nars*), oxidoreduction/antioxidant pathways (e.g., *Nqo1*, *Txnrd1,* and *Prdx5*), lysosome biogenesis, vesicle trafficking, 1-carbon metabolism (e.g., *Mthfd2* and *Shmt2*), and serine synthesis, ER/mitochondrial UPR, and basic-leucine zipper domain (bZIP) containing transcription factors mediating the UPR (e.g., *Chop*/*Ddit3*, *Atf4*, *Atf5,* and *Atf6*), all of which were characteristics of the ISR (21, 22) (Figure 3, A and B; Supplemental Figure 3, E and F; and Supplemental Data Set 1). In concert, Adenoviral-mediated *Fis1* overexpression in hepatocytes increased the protein levels of phospho-Gcn2 (an ISR upstream signaling) and Ddit3 (ISR downstream effector) compared with control hepatocytes (Supplemental Figure 3G). Ad-Fis1 also upregulated LC3B and p62 proteins. Steady-state metabolite analyses showed that the level of glyceraldehyde 3-phosphate (G3P), fumarate, and several amino acids (e.g., serine, glycine, phenylalanine, and tyrosine) were increased in Ad-Fis1 hepatocytes (Supplemental Figure 3H, and Supplemental Data Set 2). Fumarate that could be converted from phenylalanine and tyrosine has been linked to chemical-induced ISR (21). G3P is a precursor for the synthesis of serine, which, together with glycine, is involved in the 1-carbon metabolism and glutathione production (22, 27). In addition, concentrations of metabolites in the urea cycle that might be involved in detoxification during protein catabolism induced by mitophagy were higher in Ad-Fis1 hepatocytes. As 1-carbon metabolism-derived NADH/NADPH is believed to be able to contribute to ATP production and redox homeostasis (27), these results suggest that Fis1-mediated ISR promotes metabolic reprogramming, proteostasis, and redox balance.

To probe into potential downstream effectors of Fis1-induced ISR, the 5’ proximal regions of approximately 500 genes in the 16 clusters shown in Figure 3A were subjected to Homer motif analysis, which identified binding sites for the bZIP transcription factors, such as Chop/Ddit3 and Atf4 (Figure 3C, and Supplemental Data Set 3). Both Atf4 and the closely related Atf5 have been shown to mediate the transcriptional response of ISR (21, 23). In vivo, hepatic expression of *Atf5*, but not *Atf4*, was upregulated and downregulated by Ad-Fis1 and Ad-shFis1, respectively (Supplemental Figure 3, I and J), suggesting that Atf5 may be a primary downstream transcription factor of Fis1-induced ISR in the liver. In fact, adenoviral-mediated shRNA knockdown of *Atf5* (Ad-shAtf5) in primary hepatocytes reduced the expression of ISR genes compared with control scrambled shRNA (Ad-shCtl; Figure 3D, and Supplemental Figure 3K). Similar results were observed using Ad-shFis1. In addition, the induction of ISR genes (e.g., *Ddit3* and *Asns*) by Fis1 was blunted with *Atf5* knockdown (Figure 3E, and Supplemental Figure 3L). These findings support the notion that Atf5 functions as a downstream effector of Fis1-mediated ISR in the liver.

### Hepatic Fis1-Atf5 signaling suppresses metabolic inflammation.

**Figure 4:**
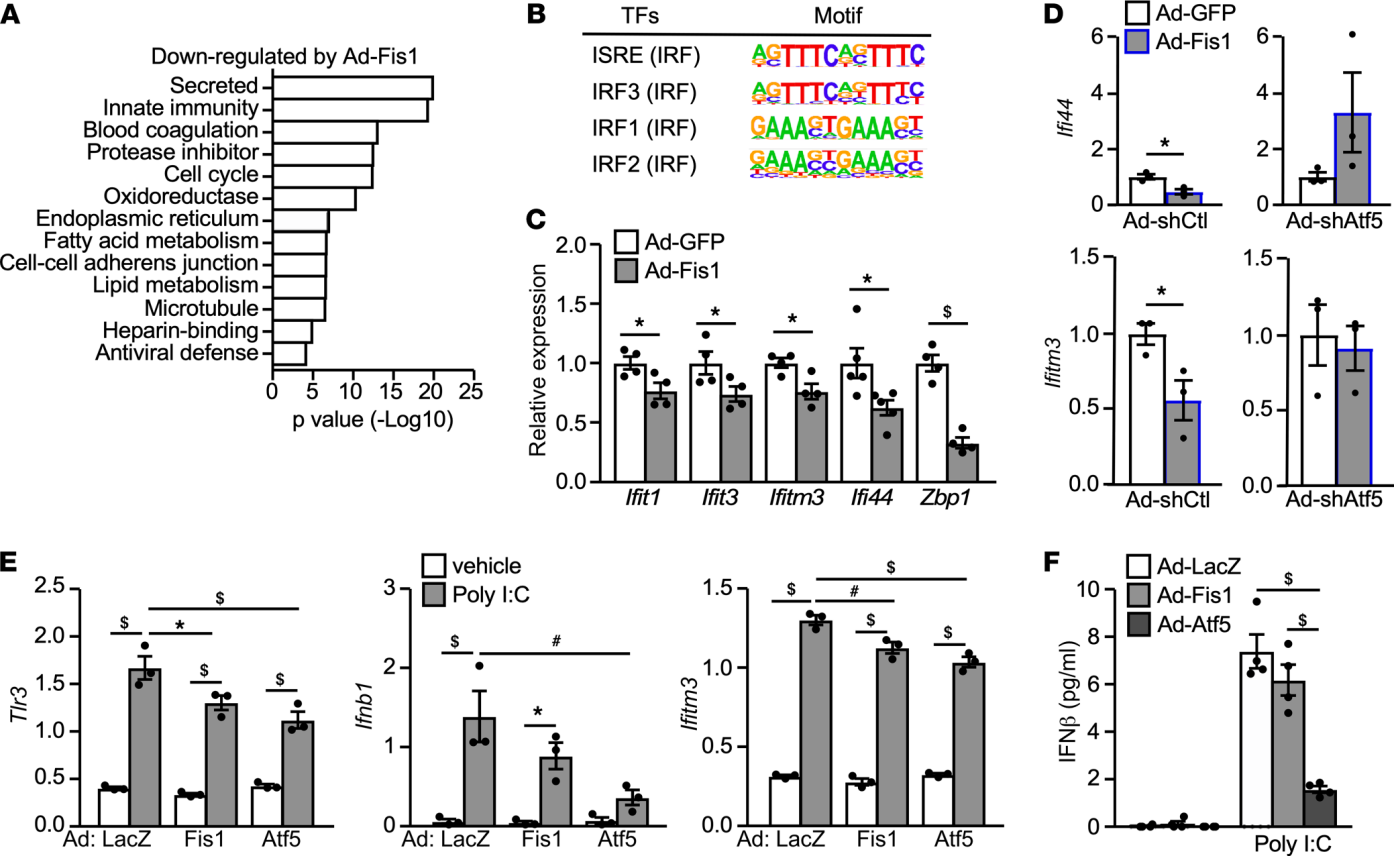
The Fis1-Atf5 axis of the integrated response suppresses IFN-I signaling. (**A**) Functional clustering analysis of 1384 genes downregulated (FDR *P* < 0.001) by Ad-Fis1 versus Ad-LacZ in primary hepatocytes identified by RNA-Seq. *n* = 4 in 1 experiment. (**B**) HOMER motif analysis to identify potential transcription factor binding sites on promoters of Ad-Fis1 downregulated genes. (**C**) Relative expression of IFN-stimulated genes in the liver of HFD-fed (12 weeks) mice infected with Ad-GFP or Ad-Fis1. Relative expression in Ad-GFP control was set as 1. *n* = 4-5, for 1 cohort. (**D**) Assessing Atf5 as a Fis1 downstream effector in regulating hepatic ISG expression using real-time PCR. Primary hepatocytes were infected with Ad-shCtl or Ad-shAtf5; 8 hours later, cells were washed and infected with Ad-GFP or Ad-Fis1. Hepatocytes were cultured for an additional 40 hours. *n* = 3, repeated twice. (**E**) *Tlr3*, *Ifnb1*, and *Ifitm3* gene expression (*n* = 3) and (**F**) IFN-β protein secretion (*n* = 6) in primary hepatocytes from mice fed a HFD for 6 weeks. Hepatocytes were infected with Ad-LacZ, Ad-Fis1, or Ad-Atf5 overnight, followed by transfection with/without 100 ng/well Poly I:C for 16 hours. Supernatant IFN-β concentration was normalized to cellular protein content. Experiments repeated 4 times. Values are presented as mean ± SEM. Significance of **C** and **D** were determined by unpaired, 2-tailed Student’s *t* test; and of **E** and **F** by 1-way ANOVA followed by Holm-Šidák multiple comparisons test. *P < 0.05; ^#^*P* < 0.01; ^$^*P* < 0.001.

Among the top enriched pathways downregulated by Ad-Fis1 were innate immune responses, notably the antiviral defense mechanism (Figure 4A, and Supplemental Data Set 1). In concert, Homer motif analysis of Ad-Fis1 downregulated genes identified interferon-stimulated response elements (ISRE) present on promoters of ISGs (Figure 4B, and Supplemental Data Set 3). As mentioned earlier, IFN-I signaling and ISGs were upregulated in the liver of obese mice (8) (Figure 1, F and G; and Supplemental Figure 1, B and C). In line with the RNA-Seq analysis, Ad-Fis1 suppressed ISGs, such as *Ifit1*, *Ifit3*, *Ifitm3*, *Ifi44*, and *Bzp1* in the liver of HFD mice, compared with the control animals (Figure 4C). In primary hepatocytes, *Atf5* knockdown abolished the ability of Fis1 to suppress the expression of *Ifitm3* and *Ifi44* (Figure 4D; Ad-GFP/Ad-Fis1 in Ad-shCtl versus Ad-shAtf5), suggesting that Atf5 could also mediate the effect of Fis1-induced ISR on modulating IFN-I signaling.

To examine the potential role of the Fis1-Atf5 axis in the activation of metabolic inflammation, the activities of Fis1 and Atf5 were acutely increased in primary hepatocytes using Ad-Fis1 or Ad-Atf5, followed by poly I:C treatment (Supplemental Figure 4A). Ad-Atf5 and Ad-Fis1 similarly downregulated the expression of *Tlr3*, *Ifnb1*, and *Ifitm3* and suppressed the production of IFN-β (Figure 4, E and F). Atf5 has been shown to localize to mitochondria and nucleus in HeLa cells (23). Because none of the commercial antibodies tested showed specific signals against endogenous or exogenous Atf5 protein, we constructed expression vectors for mouse *Atf5* with 1 copy of the HA tag at the C-terminus and established Hepa1–6 cells (a mouse hepatoma cell line) stably expressing the HA-tagged Atf5 (Hepa-Atf5) or the control vector (Hepa-Ctl). Atf5 was found primarily in the nucleus (Supplemental Figure 4B). The induction of *Ifnb1* and *Ifna4* genes by Poly I:C was also reduced in Hepa-Atf5 cells compared with control cells (Supplemental Figure 4C). Thus, Fis1-induced ISR may improve metabolic homeostasis, in part, through suppressing IFN-I–mediated metabolic inflammation.

### Atf5-Irf3 crosstalk modulates IFN-I production in liver cells.

**Figure 5:**
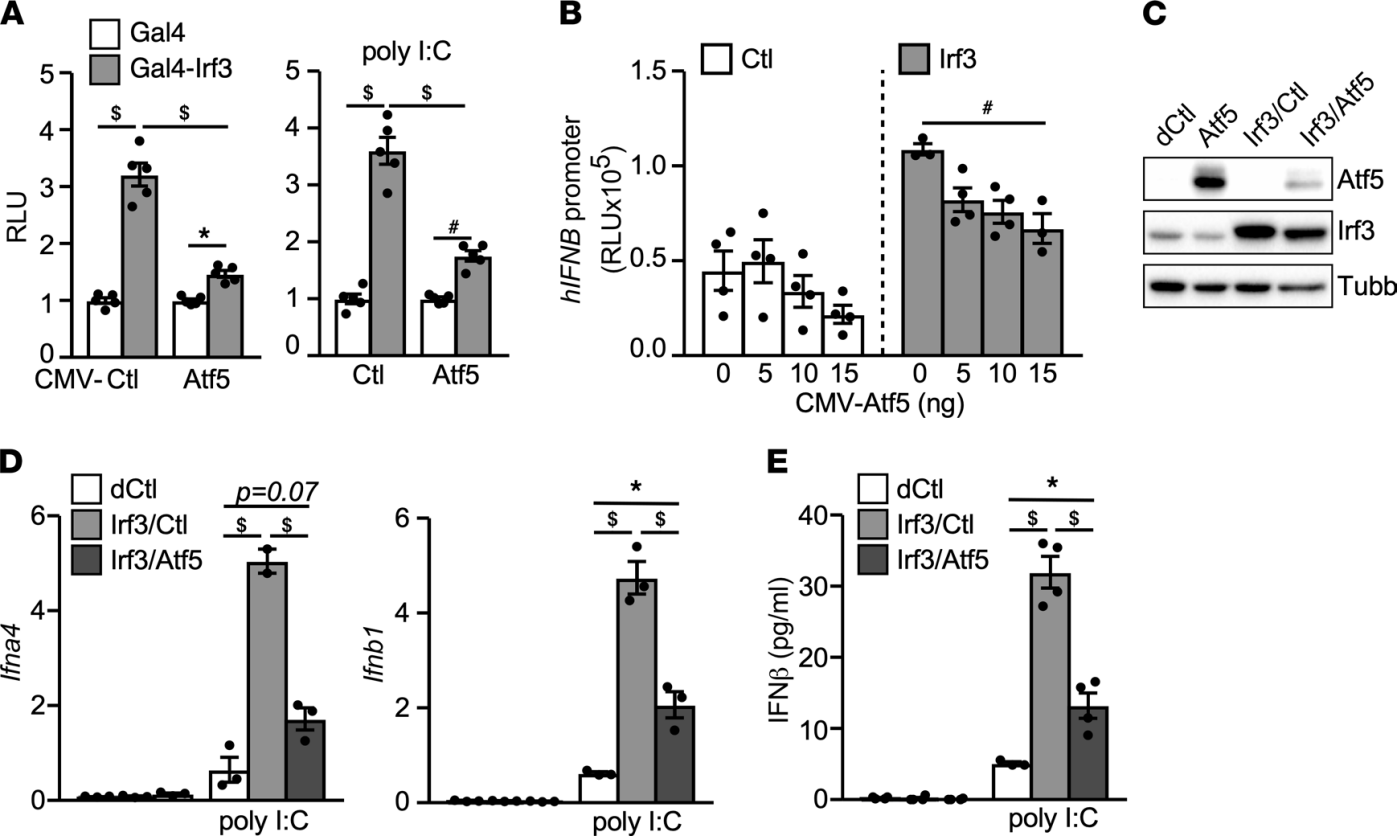
Atf5 suppresses IFN-I response through Irf3. (**A**) Atf5 inhibits the transactivation activity of Irf3. Hepa1–6 cells were cotransfected with a luciferase reporter driven by SV40 promoter with 4 copies of the Gal4-binding site and an expression vector for Gal4 or Gal4-Irf3, together with a CMV control (Ctl) or CMV-Atf5 expression vector. CMV-β-galactosidase was included to monitor the transfection efficiency. Cells were transfected with/without 100 ng/well Poly I:C for the last 16 hours. The luciferase activity was normalized by the β-galactosidase activity. RLU was presented as fold change of Gal4-Irf3 versus Gal4. *n* = 5. (**B**) Control or Irf3 overexpressing Hepa1–6 stable cell lines were cotransfected with human *IFNβ* promotor reporter, CMV-β-galactosidase, and either the CMV control or increasing amounts of CMV-Atf5 expression vector (total amount of plasmid DNA was kept same with the control vector). The luciferase activity was normalized by the β-galactosidase activity to determine the RLU. *n* = 5. (**C**) Immunoblotting showing the protein level of Atf5 (probed with anti-HA antibody) and Irf3 in Hepa1–6 “dual” stable lines expressing control empty vector (dCtl), *Irf3* (Irf3/Ctl), or *Irf3* together with *Atf5* (Irf3/Atf5). Anti-Irf3 antibody detected both endogenous and overexpressed Irf3 protein. Tubb protein level was loading control. The *Atf5* expressing Hepa1–6 “single” stable line (Supplemental Figure 4B) was included for comparison. (**D**) *Ifna4* and *Ifnb1* gene expression and (**E**) IFN-β protein secretion in control, *Irf3,* and *Irf3*/*Atf5* overexpressing Hepa1–6 stable lines stimulated with/without 100 ng/well Poly I:C for 16 hours. *n* = 3. Experiments repeated 3 times. Values are presented as mean ± SEM. Significance was determined by 1-way ANOVA followed by Holm-Šidák multiple comparisons test. *P < 0.05; ^#^*P* < 0.01; ^$^*P* < 0.001.

Homer motif analysis also identified binding sites for IRFs (Figure 4B). Notably, Irf3 is activated in response to upstream viral DNA/RNA sensing signals and the TLR3 agonist poly I:C to regulate the production of IFN-Is (6). We sought to examine whether Atf5 could block IFN-I production through crosstalk with Irf3. Expression vectors for HA-tagged *Atf5* and N-terminal FLAG-tagged mouse *Irf3* were cotransfected into AD293 cells. Coimmunoprecipitation experiments demonstrated that Atf5 could be pulled down by Irf3 (Supplemental Figure 5A). Using a Gal4 binding site-containing luciferase reporter, we showed that Gal4-Irf3 fusion protein, when tethered to the promoter, increased the reported activity in AD293 cells (Supplemental Figure 5B). Cotransfection with the *Atf5* expression vector suppressed Gal4-Irf3 transactivation activity in the absence or presence of poly I:C. Similar results were observed in Hepa1–6 cells (Figure 5A). In addition, the activity of a luciferase reporter driven by the human *IFNB* promoter was higher in Hepa1–6 cells stably expressing *Irf3* compared with control cells, which was inhibited by Atf5 co-transfection (Figure 5B). To assess the Atf5-Irf3 crosstalk on endogenous gene regulation, we established Hepa1–6 “dual” stable lines expressing control vector (Hepa-dCtl), *Irf3* with control vector (Hepa-Irf3/Ctl) or *Irf3* together with *Atf5* (Hepa-Irf3/Atf5) using a higher drug selection concentration (Figure 5C). *Irf3* overexpression substantially potentiated the expression of *Ifna4* and *Ifnb1* genes induced by poly I:C compared with control Hepa-dCtl cells (Figure 5D). This effect was blunted by *Atf5* coexpression (Hepa-Irf3/Ctl versus Hepa-Irf3/Atf5). Consistent with the decreased *Ifnb1* expression, the production of IFN-β by poly I:C stimulation was reduced in Hepa-Irf3/Atf5 cells (Figure 5E). These results suggest that Atf5 inhibits IFN-I signaling by targeting Irf3 transcriptional activity.

### Increased hepatic Atf5 activity improves glucose homeostasis.

**Figure 6:**
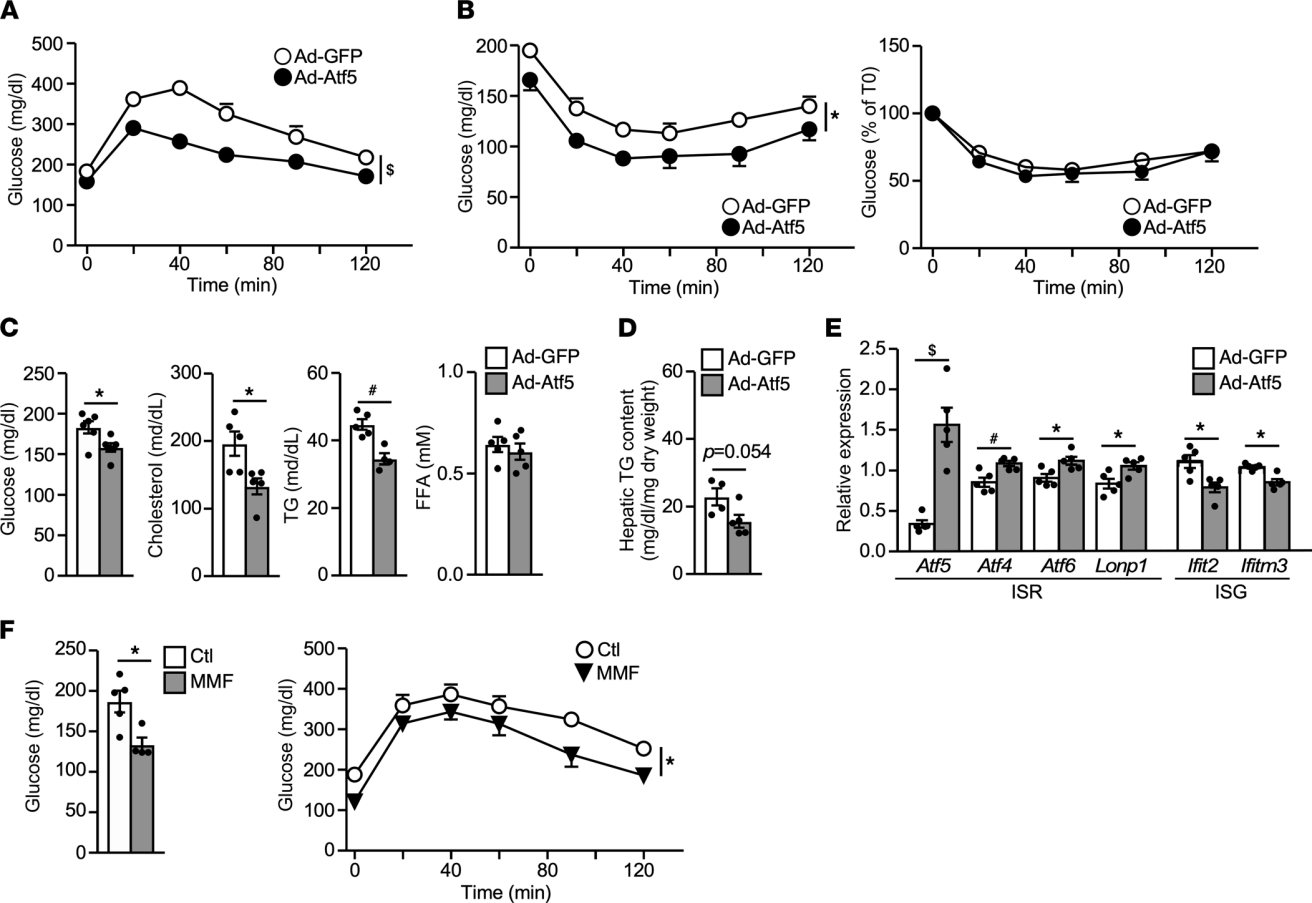
Hepatic Atf5 overexpression promotes metabolism homeostasis. (**A**) GTT and (**B**) ITT of HFD-fed male mice (12 weeks HFD) infected with Ad-GFP (control) or Ad-Atf5. *n* = 6. (**C**) Levels of fasting glucose (16-hour fasting) and serum lipids (4-hour fasting), (**D**) hepatic TG content, and (**E**) liver ISR and ISG gene expression in Ad-GFP and Ad-Atf5–infected mice. *n* = 5–6. (**F**) GTT of male mice on a HFD supplemented with vehicle or MMF (45 mg/Kg body weight, mixed in the HFD) for 6 weeks. Left panel: 16-hour fasting blood glucose. *n* = 4–5. Experiments repeated in 2 separate cohorts. Values are presented as mean ± SEM. Significance was determined by 2-way ANOVA for GTT and unpaired, 2-tailed Student’s *t* test for 2-group comparisons. *P < 0.05; ^#^*P* < 0.01; ^$^*P* < 0.001.

Given Atf5’s role in Fis1-induced ISR, we next sought to determine the effect of increased Atf5 activity in hepatic glucose and lipid metabolism. Mice on an HFD for 12 weeks were infected with Ad-Atf5 or Ad-GFP control and metabolic studies were conducted the following week. Acute *Atf5* overexpression in the liver significantly improved systemic glucose homeostasis, based on GTT and ITT (Figure 6, A and B, and Supplemental Table 3). Levels of fasting glucose, TGs, and cholesterol were also lower in Ad-Atf5 mice (Figure 6C). There was a trend toward a decrease in hepatic TG content in Ad-Atf5 mice, compared with Ad-GFP control mice (Figure 6D). In addition, Ad-Atf5 upregulated mitochondrial ISR genes and downregulated ISGs in the liver (Figure 6E). As mentioned earlier, CD8^+^ T cells are thought to be a primary target of the elevated IFN signaling that propagates metabolic inflammation in the liver of HFD mice. CD8^+^ cells were isolated from liver lysate through negative selection. The expression of ISGs, such as *Ifitm3* and *Ifi44*, in the CD8^+^ T cell population was downregulated in Ad-Atf5 mice compared with Ad-GFP control mice (Supplemental Figure 6, A and B). A similar effect was observed with hepatic *Fis1* overexpression.

A previous report has implicated a genetic link between fumarate hydratase 1 (Fh1) and ISR and shown that fumarate treatment induces several ISR genes in HeLa cells (21). The accumulation of fumarate in Ad-Fis1 hepatocytes prompted us to determine whether fumarate could modulate the activity of Atf5. In fact, treatment with monomethyl fumarate (MMF), a cell permeable derivative of fumarate, increased the transactivation activity of Atf5 when tethered to the Gal4 binding site containing reporter through a Gal4-Atf5 fusion protein in Hepa1–6 cells (Supplemental Figure 6C). MMF dose-dependently induced the expression of *Ddit3* that was partially abolished when *Atf5* was knocked down via Ad-shAtf5 in primary hepatocytes (Supplemental Figure 6D). Last, HFD-fed mice supplemented with MMF (45 mg/kg body weight) for 6 weeks lowered fasting glucose and improved glucose tolerance (Figure 6F). There was no difference in ITT or circulating lipid concentrations (Supplemental Table 4 and data not shown). Collectively, these studies indicate that the Fis1-Atf5 axis of the mitochondrial IRS protects against overnutrition-induced metabolic dysregulation.

## Discussion

In the current study, we have identified Fis1 as an endogenous signaling molecule integrating mitophagy and mitochondrion-initiated ISR. In HFD-fed mice, overnutrition sustains oxidative stress in the liver that is accompanied by defective mitophagy and enhanced IFN-I response. Transient *Fis1* overexpression in the liver promotes mitophagy, reduces oxidative damage, restores mitochondrial function, and improves glucose homeostasis. RNA-Seq analysis demonstrates that increased Fis1 activity in hepatocytes induces the entire transcriptional program of ISR and downregulates that of IFN-I signaling, in part, through Atf5. Notably, Atf5 suppresses the production of IFN-Is in hepatocytes by inhibiting the transactivation activity of Irf3, which is a key regulator of IFN-I production. IFN-Is, such as IFN-α and IFN-β, have been shown to increase mROS to inhibit autophagosome, causing mitochondrial DNA/RNA leakage that can further activate the IFN-I response (26). As such, the Fis1-Atf5 axis of the ISR may provide a therapeutic target to disrupt a detrimental feedback loop of oxidative damage, defective mitophagy, and IFN-I–induced metabolic inflammation in overnutrition.

Although mitochondrial dysfunction is known to associate with metabolic diseases in the context of obesity, whether it is a cause or consequence of metabolic dysregulation is still under investigation. Our results suggest that HFD feeding triggers unresolved oxidative stress and reduced mitophagy capacity. As mitophagy is a means to maintain mitochondrial quality control (10), defective mitophagy leads to further accumulation of damaged mitochondrial components. In addition, incomplete degradation of mitochondria could cause cytosolic mitochondrial DNA/RNA leakage (26), which is sensed by the innate antiviral immune system to activate IFN-I signaling. In fact, hepatocytes from HFD-fed mice exhibit an enhanced response to poly I:C-stimulated IFN-β production, compared with hepatocytes from NC-fed mice (Figure 1). IFN-β, in turn, inhibits mitophagy (Figure 1H), thereby initiating a vicious cycle. Previous work has found elevated IFN-I signaling in the liver of obese mice and humans that is thought to promote the accumulation of CD8^+^ T cells (8). These proinflammatory T cell subsets contribute to the development of fatty liver and glucose intolerance in HFD-fed mice. Our data indicate that hepatocytes are a potential source of IFN-Is upstream of the CD8^+^ T cell-mediated metabolic inflammation and that defective mitophagy in obesity may hinder the activation of the protective ISR to modulate the IFN-I response. In support of this notion, genes in the IFN-I signaling pathway are downregulated in CD8^+^ T cells by hepatic overexpression of *Fis1* or *Atf5*.

Fis1 has recently been shown to play an essential role in autophagosome formation during mitophagy through interaction with a mitochondrial Rab GTPase-activating protein TBC1D15 as well as LC3 (25). It has also been shown to act in sequence with fission factors to couple oxidative stress-induced fission with the downstream mitophagy process (24). Consistent with these observations, knockdown of Fis1 in hepatocytes blocks paraquat-induced mitophagy (Supplemental Figure 2C). In addition, enhanced and reduced Fis1 activities in the liver are associated with decreased and increased mROS levels, respectively (Figure 2H, and Supplemental Figure 2B), supporting an important role for Fis1-mediated mitophagy in managing oxidative stress. Fis1 activity is also inversely correlated with glucose intolerance induced by HFD feeding. At the molecular level, hepatic Fis1 could modulate metabolic homeostasis through multiple pathways of the ISR, such as antioxidant defense mechanism, redox homeostasis, and proteostasis. Therefore, in addition to managing oxidative stress, Fis1 sustains cellular function by connecting 2 mitochondrial quality control mechanisms, mitophagy and ISR.

RNA-Seq analysis reveals that Fis1 activation suppresses IFN-I signaling, which has not been described as part of the ISR. The discrepancy is likely due to differences in the trigger and cell system, as previous studies employ chemical inhibitors in immortalized cells (21). As described earlier, elevated IFN-I signaling in the liver, including the expression of *IRF3*, has been shown to correlate with fatty liver diseases in humans and glucose intolerance and dysregulated hepatic metabolism in mice (8). We have identified Atf5 as a downstream transcriptional factor mediating not only the expression of several ISR genes but also the inhibition of IFN-I signaling. Our results suggest that Atf5 suppresses the transactivation activity of Irf3 through physical interaction. Overexpression of *Fis1* or *Atf5* in hepatocytes is sufficient to blunt IFN-β production by poly I:C stimulation, which activates Irf3. As such, the Fis1-Atf5 axis could also promote metabolic homeostasis through inhibition of IFN-I signaling.

A limitation of the current study is the overexpression nature of adenovirus-mediated hepatic gene delivery. However, this approach allows transient activation (or inhibition) of Fis1-mediated mitophagy/ISR without potential compensatory effects of mouse genetic models. In addition, an unanswered question is how Fis1 induces Atf5 activity. Although a previous report indicates that Atf5 translocates from mitochondria to the nucleus in HeLa cells upon UPR^mt^ (23), Atf5 appears to be primarily localized to the nucleus in liver cells. Prior work has demonstrated that fumarate is able to induce several ISR genes (21). Our metabolite analysis shows that fumarate accumulates in *Fis1* overexpressing hepatocytes. MMF, a fumarate derivative, increases Atf5 activity in the report assay and its ability to upregulate *Ddit3* in hepatocytes is partially dependent on Atf5 (Supplemental Figure 6, C and D), suggesting that fumarate may serve as a signaling molecule linking Fis1-induced ISR and Atf5 activation. Similar to hepatic Atf5 activation, MMF treatment improves glucose tolerance in HFD-fed mice. MMF is an approved drug to treat relapsing forms of multiple sclerosis (28). The proof-of-principle study presented in the current work demonstrates the possibility of developing small molecules targeting the Fis1-Atf5 axis of ISR to treat metabolic diseases.

## Methods

### Animal studies.

Mouse strains used in all experiments were in the C57BL/6J background (JAX). Animals were housed at 22°C in a barrier facility and kept on a 12-hour light, 12-hour dark cycle with free access to water and food except for the fasted/refed manipulation. For metabolic studies, mice were fed a NC (Rodent Diet 20 5053, PicoLab) or HFD (Fat Calories 60% F3282, Bio-Serv) for 4–12 weeks starting at 6 weeks of age. Experiments were performed in male mice, while primary hepatocytes were derived from both male and female mice, which yielded similar results. For glucose (1.5 g/kg body weight) and insulin (1 U/kg) tolerance tests, mice were fasted for 16 hours (except Figure 2B, which was 4-hour fasted) and 4 hours, respectively. Blood glucose was measured before and 20, 40, 60, 90, and 120 minutes following the glucose/insulin injection. Serum TG (Infinity, TR22421), free cholesterol (Wako, 999-02601), nonesterified fatty acid (Wako, NEFA-HR2), and insulin (Meso Scale Discovery, K152BZC-1) were measured according to manufacturers’ instructions. For measurement of liver TG content, tissues were homogenized in buffer containing 50 mM Tris, 100 mM NaCl, and 0.1% NP40. Tissue was dried using a speed-vac centrifuge and dry tissue weight was used for normalization. Lipids were extracted with chloroform and dried in a fume hood. Protein carbonylation to assess oxidative damage using liver lysates was conducted with protein carbonyl content assay kits (Abcam, ab126287).

### Adenovirus-mediated gene expression.

Adenovirus was amplified in AD293 cells, which were maintained in Dulbecco’s Modified Eagle medium (DMEM, Corning) containing 4.5g/L glucose and 10% FBS (Gemini Bio-Products). Virus was purified by CsCl gradient, followed by dialysis against PBS overnight. Ad-GFP, Ad-LacZ, Ad-Cox8-GFP, Ad-Cox8-mCherry, and Ad-Fis1 were generated using the Ad-Easy Adenoviral Vector System. The rest of the viruses were purchased from Vector BioLabs: Ad-Atf5 (ADV-253209), Ad-Dmn1l (ADV-257347), Ad-shCtl (1122N: scrambled shRNA control), Ad-shFis1 (shADV-259434), and Ad-shAtf5 (shADV-253209). In vivo adenoviral-mediated hepatic overexpression/knockdown was conducted by retro orbital injection (~5 × 10^10^ PFU/mouse). Subsequent metabolic characterizations were carried out 3–8 days following injection. For ex vivo studies, primary hepatocytes were isolated using collagenase digestion (Liberase, Roche) through portal vein perfusion and a 45% Percoll (GE Life Sciences) gradient was used to separate live and dead hepatocytes (29). Primary hepatocytes were maintained in William’s E Medium containing 5% FBS and 2 mM l-glutamine, after which 2 × 10^5^ cells/well were seeded into 12-well plates (Corning Life Science) overnight, followed by adenovirus infection (MOI 1–10) for 32–48 hours. To assess the effect of Atf5 knockdown on Fis1 activity, hepatocytes were first infected with Ad-shCtl or Ad-Atf5. After 8 hours, each set of cells was washed and infected with Ad-GFP or Ad-Fis1 for an additional 40 hours.

### CD8^+^ T cell enrichment.

Mice (15 weeks old, male, C57BL/6J) fed an HFD for 2 months were infected with Ad-GFP, Ad-Fis1, or Ad-Atf5. Five days after infection, livers (*n* = 2) were collected, pooled, and mechanically dissociated to release hepatocytes and immune cells. Cell suspension was filtered through a 70 μm cell strainer, washed with red blood cell lysis buffer and separated in 40% Percoll gradient to remove hepatocytes. Leucocytes were further purified through Ficoll-Paque gradient. CD8^+^ cells were enriched using BD IMag Mouse CD8^+^ T Lymphocyte Enrichment Set (BD Bioscience, 558471), which employed a negative selection method that pulled down CD8^–^ immune cells with magnetic beads conjugated with an antibody cocktail, followed by stimulation with phorbol 12-myristate 13-acetate (100 ng/mL, Sigma) and ionomycin (1 μg/mL, Sigma) for 3 hours. RNA extraction was performed in both the bead and flow-through (CD8^+^ T cell-enriched) fractions to assess the purification efficiency and the expression of ISGs.

### RNA-Seq and data analysis.

RNA-Seq was performed on RNA from 4 cell culture replicates per treatment. Sequencing and raw data processing were conducted at the IMB Genomics Core and IMB Bioinformatics Service Core at the Academia Sinica (Taipei, Taiwan). Briefly, samples were quantified with Ribogreen (Life Technologies) and RNA integrity was checked with a Bioanalyzer 2100 (Agilent). (RIN > 8; OD 260/280; and OD 260/230 > 1.8) RNA libraries were prepared with the TruSeq Stranded mRNA Library Preparation Kit (Illumina) according to the manufacturer’s instructions using 2 μg of RNA per sample. Sequencing was performed using an Illumina NextSeq 500 High Output Kit (75 cycles, 400 million total reads) on an Illumina NextSeq 500 instrument. Raw data was processed using the Qiagen CLC Genomics Workbench (v.10.1.1). Raw sequencing reads were trimmed by removing adapter sequences, low-quality sequences (Phred quality score of < 20), and sequences with lengths greater than 25 bp. Quality control for individual samples were examined. Sequencing reads were mapped to the mouse genome assembly (mm 10) from the University of California, Santa Cruz with the following parameters: mismatches = 2, minimum fraction length = 0.9, minimum fraction similarity = 0.9, and maximum hits per read = 5. Normalization and calculation of expression values were performed using the Differential Gene Expression for RNA-Seq Tool in the Qiagen CLC Genomics Workbench v10. Normalization of gene expression was based on transcripts per kilobase million (RPKM). Statistical analysis of differential gene expression was calculated by generalized linear model implemented in the *EdgeR* package in R, accounting for differences in library size between samples (30). A significance cutoff for differentially expressed genes of *FDR* < 0.001 was used to determine differentially expressed genes. RNA-Seq data have been deposited in the Gene Expression Omnibus database under the accession number GSE169630. Gene Ontology (GO) analyses were performed with DAVID (https://david.ncifcrf.gov). De Novo Motif Enrichment was performed with HOMER (http://homer.ucsd.edu/homer) searching 200 bp upstream and downstream of transcription start sites with default settings. To identify downstream transcription factors mediating Fis1-upregulated genes in the ISR, approximately 500 genes in the 16 clusters shown in Figure 3A were analyzed. For suppressed pathways, all 1384 genes downregulated by Fis1 were analyzed.

### Metabolomics analysis.

Untargeted metabolomics analysis using gas chromatography–time-of-flight mass spectrometry was conducted by the West Coast Metabolomics Center at the University of California, Davis. In brief, 10 million cells were lifted, pelleted, and washed twice with PBS for each of the 4 replicates. Cell lysates were homogenized by metal bead beating, and metabolites were extracted using 80% methanol. Following extraction, cell pellets were solubilized in Tris-HCl Urea buffer (pH 8.0) containing 1% SDS to measure cellular protein content. All metabolite readings were normalized to total protein content.

### Mitochondrial assays.

Mitochondria were isolated from primary hepatocytes or liver by differential centrifugation. In brief, cells were resuspended in 500 μL of ice-cold cell mitochondrial isolation buffer consisting of 70 mM sucrose, 50 mM Tris, 50 mM KCl, 10 mM EDTA, and 0.2% fatty-acid free BSA (pH 7.2) and extruded through 29-gauge syringes 20 times. Lysates were spun at 800*g* to pellet nuclei, and supernatants were spun at 8,000*g* to isolate mitochondria. For liver, a piece of tissue about 0.2 g was washed twice in ice-cold tissue mitochondrial isolation buffer consisting of 70 mM sucrose, 210 mM mannitol, 5 mM HEPES, 1 mM EDTA, and 0.2% fatty acid free BSA. Tissue pieces were minced with surgical scissors into small pieces, broken up in the Dounce homogenizer 5 times, and spun at 800*g* to pellet nuclei. The supernatant was spun at 8000*g* to isolate mitochondria. Pelleted mitochondria were washed once more with 500 μL mitochondrial isolation buffer. To measure ROS production in isolated mitochondria, 15 μg of mitochondria were resuspended in 500 μL mitochondrial isolation buffer containing 5 μM MitoSox Red and 100 μM MitoTracker Green FM with 10 mM sodium succinate. Mitochondria were incubated for 20 minutes at 37°C, washed with mitochondrial isolation buffer, and resuspended for flow cytometry. Mitochondria were identified by forward scatter (FSC) and side scatter (SSC) with positive MitoTracker Green staining and the MFI of MitoSox Red staining was determined. Electron flow assays with isolated mitochondria were performed as described previously (17). Isolated mitochondria (50 μg/well) were plated in XF24 microplates in buffer containing 70 mM sucrose, 220 mM mannitol, 10 mM KH_2_PO_4_, 5 mM MgCl_2_, 2 mM HEPES, 1 mM EGTA, and 0.2% BSA. Initial assay buffer additionally contained 10 mM pyruvate, 2 mM malate, and 6 μM FCCP for complex I-driven respiration. Sequential injections of 2 μM rotenone, 10 mM succinate, 4 μM antimycin a, and 100 μM TMPD/10 mM ascorbate were used to measure complex II and IV respiration.

### Mitochondrial image acquisition and analysis.

For in vivo mitochondrial network analysis, mice infected with Ad-Cox8-GFP were perfused with 10% formalin. Liver samples were further processed in 30% sucrose solution and embedded in OCT for cryosectioning. For ex vivo mitochondrial network analysis, hepatocytes seeded on coverslips were infected with Ad-Cox8-mCherry for 8 hours, followed by a second virus infection for another 40 hours. Cells were then switched to the EBSS medium (Gibco, 14155063) containing 0.5 mM MgCl2, 1.8 mM CaCl2, and 1 g/L glucose for 4 hours and fixed with 4% paraformaldehyde for 10 minutes. Slides were mounted with mounting media containing DAPI to stain nuclei. All images were acquired by confocal microscopy and analyzed by ImageJ software (NIH). The image of GFP channel is subjected to default “Moments_Thresholding” to measure mitochondrial area and perimeter.

### Plasmid construction and luciferase reporter assay.

Full-length cDNAs encoding mouse *Atf5* (with a C-terminal HA tag, pCMV-Atf5-HA) and *Irf3* (with a N-terminal FLAG tag, pCMV-FLAG-Irf3) were cloned into pCMV and pCMV-Gal4 expression vectors or pBabe-puro retroviral vector for Hepa1–6 stable line generation. The Gal4 binding site (4 copies)-containing reporter was driven by the SV40 promoter. IFN-β pGL3 luciferase reporter was from Addgene. For transient transfection in AD293 cells and Hepa1–6, 1 × 10^4^ cells/well in 96-well plates were seeded overnight. Then, 50 ng luciferase reporter and 5 ng expression vectors were cotransfected with 25 ng CMV-β-galactosidase using LT1 transfection reagent for 24 hours. Cells were lysed 40–48 hours after transfection using the passive lysis buffer to measure the luciferase activity (Promega), which was normalized to the β-galactosidase activity to obtain RLU. Experiments were carried out in 4–6 replicates.

### Generation of stable Hepa1–6 cell lines.

Hepa1–6 cells were maintained in DMEM with 4.5g/L glucose and 10% FBS. For the generation of single *Atf5* or *Irf3* expressing stable lines, HA-tagged *Atf5* or FLAG-tagged *Irf3* (see Plasmid Construction & Luciferase Reporter Assay) were cloned into the pBabe-puro vector. Retrovirus carrying control (pBabe-puro empty vector), *Atf5,* or *Irf3* was produced in Phoenix packaging cells. Hepa1–6 cells were incubated with retrovirus-containing conditioned media with 4 μg/mL polybrene for 48 hours and selected with 4 μg/mL puromycin. To generate dual *Irf3/Atf5* expressing lines, Hepa1–6 cells were infected with control, control + *Irf3,* or *Atf5* + *Irf3* retrovirus and selected with 8 μg/mL puromycin. Experiments were performed after 3 passages of puromycin selection.

### IFN-I signaling studies.

The 2 × 10^5^ primary hepatocytes or Hepa1–6 cells/well were seeded in 12-well plates. Cells were stimulated with Poly I:C (Invivogene) complexed with LT1 transfection reagent (Mirus) at 100 ng/well for 16–24 hours. Conditioned media were collected for IFN-β measurement using ELISA kits (R&D) and normalized by total cellular protein amount.

### Cellular fractionation.

Nuclear fraction (NUC) was isolated by using a NE-PER kit (Thermo Fisher Scientific). Mitochondrial fraction (MIT) was isolated as described in “Mitochondrial Assays”. Mitochondria depleted cytosolic fraction (CYT) was isolated by differential centrifugation (31). In brief, cells were resuspended in 500 μL of ice-cold cell homogenized medium consisting of 75 mM sucrose, 225 mM mannitol, 30 mM Tris-HCl ph7.4, and 0.1 mM EGTA and extruded through 29-gauge syringes 20 times. Lysates were spun at 1000*g* to pellet nuclei and unbroken cells. The supernatant was spun at 8000*g* to remove mitochondria.

### Immunoblotting.

Cells were washed by PBS and lysed in cold lysis buffer (100 mM NaCl, 20 mM Tris-Cl pH8.0, 1 mM EDTA, 1 mM DTT, 0.1 % NP-40, and 10 % glycerol) with phosphatase inhibitors (0.5 mM NaF, 50 μM NaVO_4_, 100 μM Na_4_P_2_O_7_, 100 μM β-glycerophosphate, and 50 μM Na_2_MoO_4_), protease inhibitors cocktail (cOmplete, Roche) and 1 mM phenylmethylsulfonyl fluoride. Cellular protein contents were measured by BCA kit (Pierce) or Bradford reagent (BioRad), separated by SDS-PAGE and transferred to PVDF membranes (Thermo Fisher Scientific). Immunoblotting was conducted by overnight incubation with primary antibodies (Supplemental Table 5) in 1% BSA in TBST buffer. The ECL signal was imaged using a BioRad ChemiDoc XRS+ imaging system.

### Immunoprecipitation.

AD293 cells were cotransfected with pCMV-Atf5-HA and pCMV-FLAG-Irf3 for 48 hours. Cell lysates were subjected to IP with anti-flag M2 beads (Sigma-Aldrich) for 4 hours followed by immunoblotting using anti-FLAG and anti-HA antibodies.

### Gene expression.

Cellular RNA was isolated using NucleoSpin RNA Plus kit (Macherey-Nagel). Tissue RNA was extracted using Trizol. Purified RNA was reverse transcribed into cDNA using the Verso cDNA synthesis kit (Thermo Fisher Scientific). Relative gene expression was determined by real-time qPCR with SYBR Green using relative standard curves and normalized to *36b4* (*Rplp0*). Primer sequences are listed in Supplemental Table 6.

### Statistics.

All data are presented as mean ± SEM. Detailed analyses of RNA-Seq were described in “RNA-Seq and analysis”. Other statistical analyses were performed using GraphPad Prism 7. The comparison of 2 parameters was performed using 2-tailed, unpaired Student’s *t* test and 1-way ANOVA followed by Holm-Šidák multiple comparisons test was used for conditions with more than 2 parameters. Cell-based experiments were performed with 3–6 biological replicates and repeated at least 3 times, except for Figure 3E and Figure 4D, which were repeated twice, and RNA-Seq and metabolite analysis, which were done once. Animal studies were performed in 2 cohorts for each treatment, except for the 12-week HFD cohort infected with Ad-GFP and Ad-Fis1 (Supplemental Table 7). Analysis of in vivo studies (glucose and insulin tolerance tests) with multiple parameters was performed using 2-way ANOVA. Statistical significance was defined as *P < 0.05, ^#^*P* < 0.01 and ^$^*P <* 0.001.

### Study approval.

All animal studies were approved by the Harvard Medical Area Standing Committee on Animal Research.

## Author contributions

YHL, JP, NHK, KAS, ICN, and DJ conducted experiments and analyzed data. NHK and DJ assisted in assay development and reagent generation. DJ was involved in idea development. YHL and CHL designed the experiments, generated the figures, and wrote the manuscript. JP designed and conducted most experiments and generated figures for revision with help from MMC. JP was assigned the second of co–first authors with YHL, who was involved in the original concept development.

## Supplementary Material

Supplemental data

Supplemental table 1

Supplemental table 2

Supplemental table 3

## Version 1 01/11/2022

In-Press Preview

## Version 2 02/22/2022

Electronic publication
