# Supplementary material for: Hepatic Fis1 regulates mitochondrial integrated stress response and improves metabolic homeostasis
Source: JCI Insight. 2022 Feb 22;7(4):e150041. doi: 10.1172/jci.insight.150041 (PMC8876406; doi:10.1172/jci.insight.150041)
Supplement: Supplemental data [file jciinsight-7-150041-s184.pdf]

Supplemental Information

Hepatic Fis1 regulates mitochondrial integrated stress response and improves metabolic homeostasis

Yae-Huei Liou, Jean Personnaz, David Jacobi, Nelson H. Knudsen, Mayer M. Chalom, Kyle A. Starost, Israel C. Nnah, and Chih-Hao Lee

Supplemental Figures

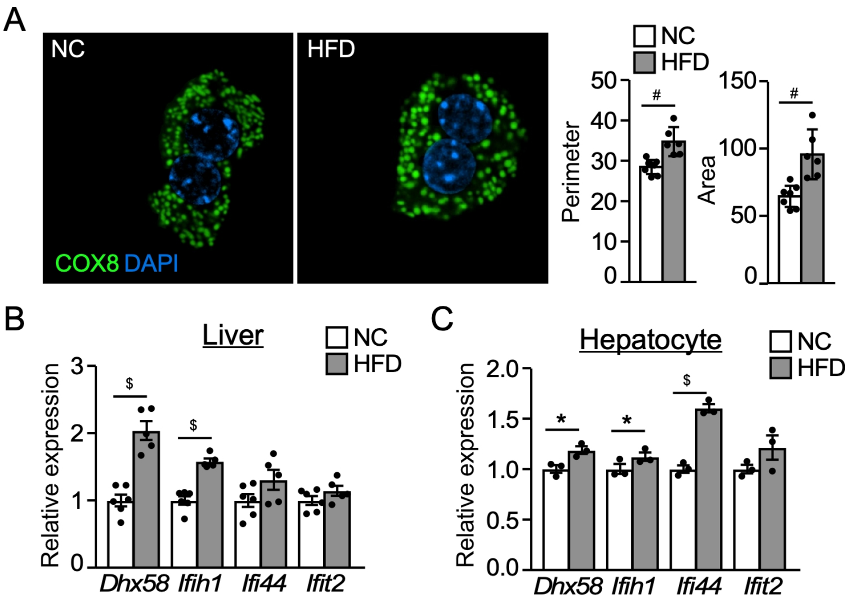

Supplemental Figure 1. Chronic over-nutrition is associated with mitochondrial dysfunction and enhanced IFN-stimulated gene expression in mouse livers. (**A**) Representative confocal images of liver cryosections (20 µm) from NC or HFD fed mice collected at ZT2 (8 am). C56BL/6J male mice were infected with Ad-Cox8-GFP to label mitochondria. Quantification of mitochondrial morphology was analyzed using ImageJ. n=6. (**B**) Relative expression of IFN-stimulated genes determined by real-time PCR in the liver and (**C**) in primary hepatocyte from male mice fed a NC or HFD for 8 weeks starting at 6-week old of age. n=5-6. Experiments repeated in 2 mouse cohorts. Values are presented as mean ± SEM. Significance was determined by unpaired, two-tailed Student's *t* test. *p<0.05, $^{\#}p$<0.01, $^{\$}p$<0.001.

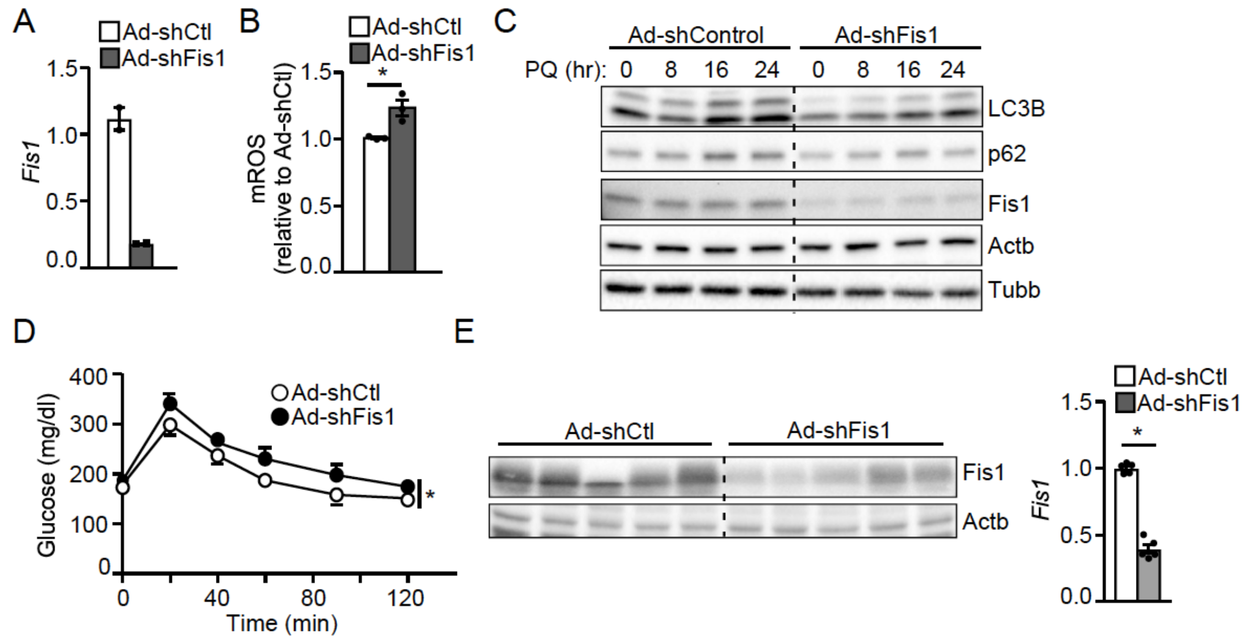

Supplemental Figure 2. Reduced hepatic Fis1 activity worsens oxidative stress and glucose intolerance. (**A**) *Fis1* gene expression by real-time PCR to assess the knockdown efficiency of Ad-shFis1 in primary hepatocytes. Ad-shCtl: scrambled shRNA control adenovirus. (**B**) Mitochondrial ROS (mROS) was measured 48 hours after Ad-shCtl and Ad-shFis1 infection in primary hepatocyte. mROS was determined using mean fluorescence intensity of the MitoSOX signal and presented as fold change of Ad-shFis1 vs Ad-shCtl. n=3. (**C**) Immunoblotting showing LC3B, p62 and Fis1 protein levels in hepatocytes infected with Ad-shCtl or Ad-shFis1 for 48 hours, followed by 0.2 mM paraquat treatment for indicated time points. Paraquat was used to trigger oxidative stress. Tubb served as a loading control. (**D**) Glucose tolerance test (GTT) in HFD fed (4 weeks) male mice infected with Ad-shCtl or Ad-shFis1. n=5. (**E**) Immunoblotting (left panel) and qPCR (right panel) showing Fis1 protein and mRNA levels in liver of HFD fed male mice infected with Ad-shCtl or Ad-shFis1. n=5. A-C: repeated 3 times and D-E:conducted in 2 separate cohorts. Values are presented as mean ± SEM. Significance was determined by Two-way ANOVA for GTT and unpaired, two-tailed Student's *t* test for two group comparisons. *p<0.05.

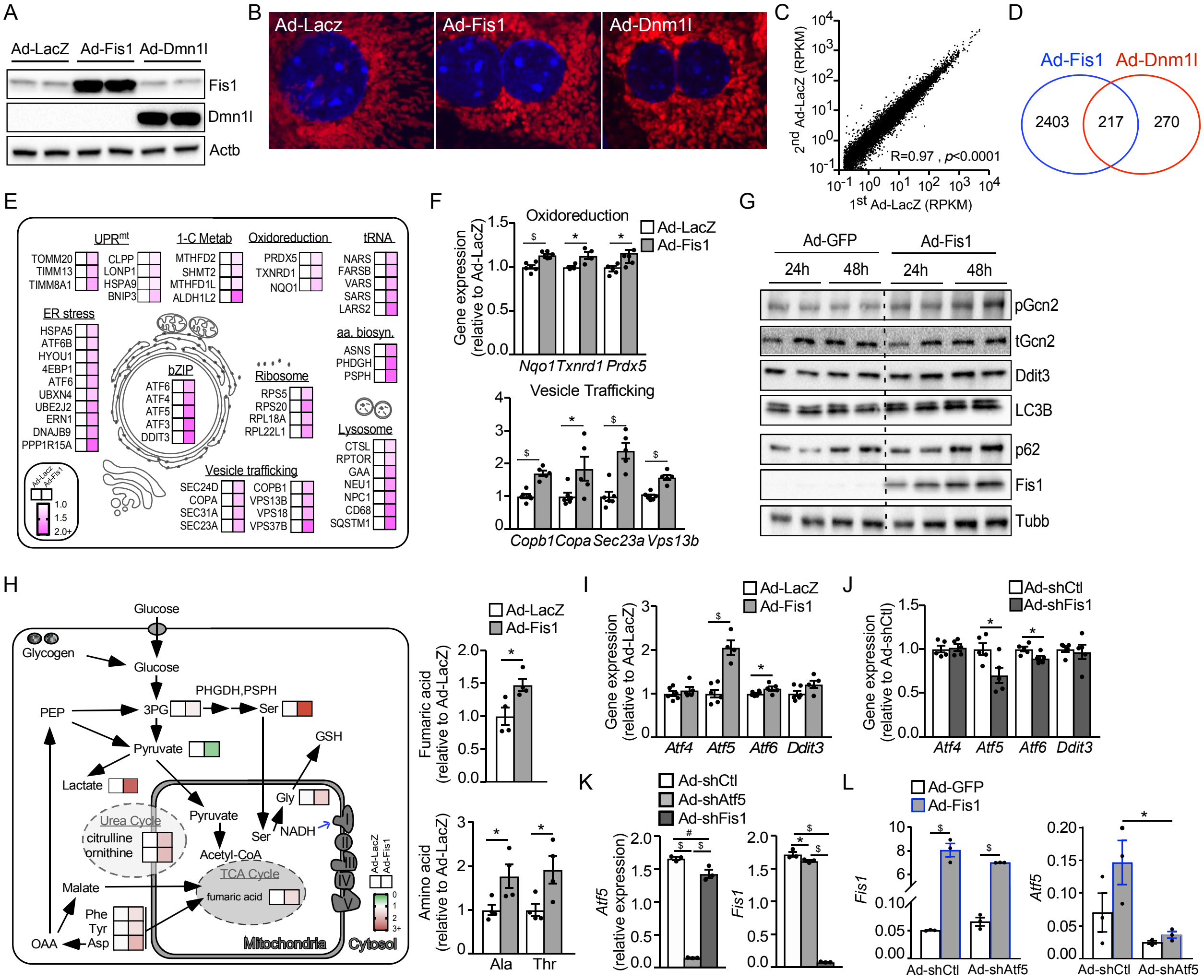

Supplemental Figure 3. Fis1 induces integrated stress response in mouse hepatocytes. (**A**) Evaluation of Fis1 and Dnm1l (Drp1) over-expression by Ad-Fis1 and Ad-Dnm1l using immunoblotting. Primary hepatocytes were infected with Ad-LacZ (control), Ad-Fis1 and Ad-Dnm1l for 48 hours. Actb protein level was used for loading control. n=2, repeated 3 times. (**B**) Mitochondrial morphology in LacZ (Ad-LacZ, control), Fis1 (Ad-Fis1) or Dnm1l (Ad-Dnm1l) over-expressing primary hepatocytes. Hepatocytes were co-infected with Ad-Cox8-mCherry to label mitochondria and cultured in the EBSS (with 1 g/L glucose) low nutrient medium to trigger fusion (elongated mitochondrial networks). Experiments repeated 3 times. (**C**) RPKM dot plot analysis of gene expression in Ad-LacZ infected hepatocytes comparing two independent RNA-seq experiments to assess potential variables contributed by different adenovirus preparations. The Pearson Correlation Coefficient r=0.97, $p<0.001$. (**D**) Venn diagram comparing genes significantly changed (FDR <0.001) by Ad-Fis1 or Ad-Dnm1l (vs Ad-LacZ) in hepatocytes identified by RNA-seq. (**E**) Heatmap showing representative genes/pathways from functional clustering of Ad-Fis1 up-regulated genes. (**F**) Validation of RNA-seq results for oxidoreduction and vesicle trafficking genes of Ad-Fis1 or Ad-LacZ infected hepatocytes by real-time qPCR. n=4-5. (**G**) Immunoblotting showing phosphorylated and total Gcn2, Ddit3, LC3B, p62 and Fis1 protein levels. Primary hepatocytes of NC fed male mice were infected with Ad-GFP or Ad-Fis1 for 24 or 48 hours. n=2, repeated twice. (**H**) Summary of metabolomic analysis (left panel) and the fold change of selected

metabolites (right panel) regulated by Ad-Fis1, compared to Ad-LacZ in primary hepatocytes. n=4 for one experiment. (**I**) Expression of stress responsive transcription factors in the liver of mice with hepatic *Fis1* over-expression (Ad-Fis1 vs Ad-LacZ, see mouse cohort in Figure 2A) or (**J**) in mice with hepatic *Fis1* knockdown (Ad-shFis1 vs Ad-shCtl, see mouse cohort in Supplemental Figure 2D). n=5. (**K**) Assessing the efficiency of knocking down *Atf5* or *Fis1* in primary hepatocytes (Ad-shFis1 or shAtf5 vs Ad-shCtl; data was associated with Figure 3D). n=3. (**L**) Relative expression of *Fis1* and *Atf5* by real-time PCR in hepatocytes infected with combinations of Ad-GFP/Ad-Fis1 and Ad-shCtl/Ad-shAtf5. Data was associated with Figure 3E and 4D. n=3. Values are presented as mean ± SEM. Significance of **F**, **H**, **I** and **J** were determined by unpaired, two-tailed Student's *t* test; **K** and **L** by One-way ANOVA followed by Sidak multiple comparisons test. $^{*}p<0.05$, $^{\#}p<0.01$, $^{\$}p<0.001$.

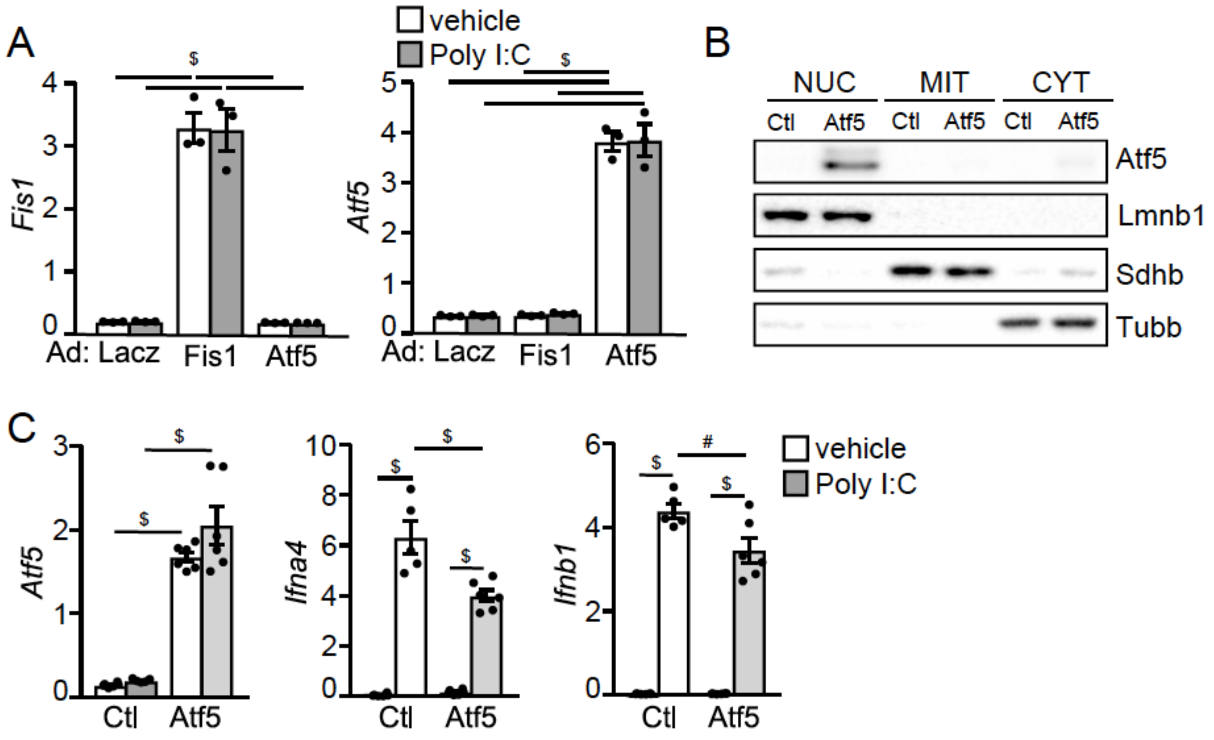

Supplemental Figure 4. Nuclear localized Atf5 regulates IFN-I response. (**A**) Assessing *Fis1* and *Atf5* genes expression in primary hepatocytes infected with Ad-LacZ, Ad-Fis1 or Ad-Atf5 with/without Poly I:C stimulation (see Figure 4E). n=3. (**B**) Immunoblotting showing cellular localization of Atf5 (with C-terminal HA-tag) in Hepa1-6 mouse hepatoma cells. Cell lysates from control (Ctl) and *Atf5* over-expressing Hepa1-6 stable lines were fractionated to obtain the nuclear (NUC), mitochondria (MIT) and cytosolic (CYT) fractions. An equal amount of protein was loaded. Anti-HA antibody was used to probe Atf5 protein. Lmnb1, Sdhb and Tubb were used to assess the purity of the nuclear, mitochondrial and cytosolic fraction, respectively. (**C**) *Atf5*, *Ifna4* and *Ifnb1* gene expression in control and *Atf5* over-expressing Hepa1-6 stable lines stimulated with/without 100 ng/well Poly I:C for 24 hours. n=5-6. Data represent 1 of the 3 repeated experiments. Values are presented as mean ± SEM. Significance was determined by One-way ANOVA followed by Sidak multiple comparisons test. $^{\#}p<0.01$, $^{\$}p<0.001$.

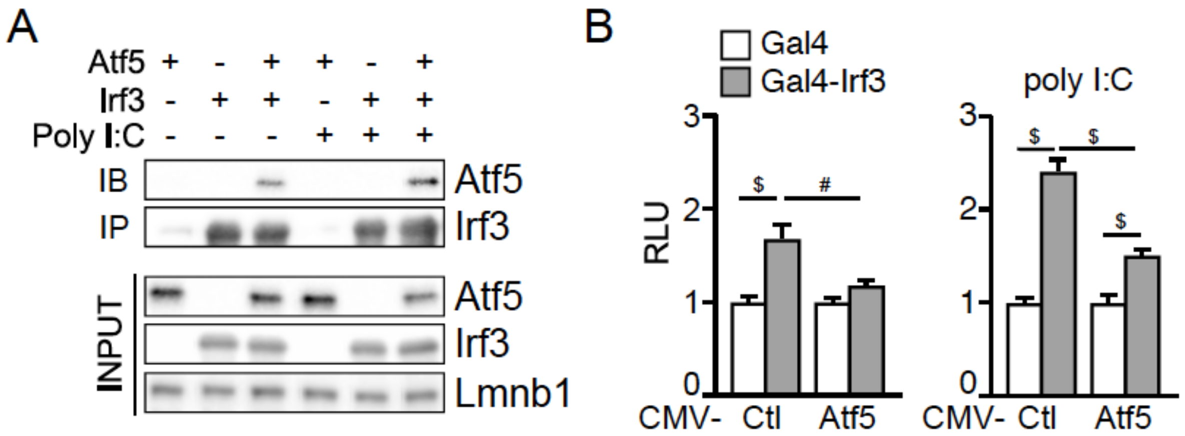

Supplemental Figure 5. Atf5 interacts with Irf3. (**A**) Immunoblotting showing co-immunoprecipitation of Atf5 with Irf3. AD293 cells were transfected with expression vectors for Atf5 (CMV-Atf5-HA), Irf3 (CMV-FLAG-Irf3) or Atf5/Irf3. Thirty-two hours later, cells were stimulated with/without 100 ng/well Poly I:C for an additional 16 hr. IP: immunoprecipitation with anti-FLAG M2 affinity gel; IB: immunoblotting with anti-HA antibody; Input: 1/20 of the cell lysate used for IP. Lmnb1 was used for loading control. (**B**) Atf5 suppresses the transactivation activity of Irf3. AD293 cells were co-transfected with a luciferase reporter driven by SV40 promoter with 4 copies of the Gal4-binding site and an expression vector for Gal4 control or Gal4-Irf3, together with a control (Ctl) or *Atf5* expression vector (driven by CMV promoter). CMV-β-galactosidase was included to monitor the transfection efficiency. Cells were transfected with/without 100 ng/well Poly I:C for the last 16 hours. The luciferase activity was normalized by the β-galactosidase activity. Relative light unit (RLU) was presented as fold change of Gal4-Irf3 vs Gal4. n=5. Data represent 1 of the 3 repeated experiments. Values are presented as mean ± SEM. Significance was determined by One-way ANOVA followed by Sidak multiple comparisons test. $^{\#}p<0.01$, $^{\$}p<0.001$.

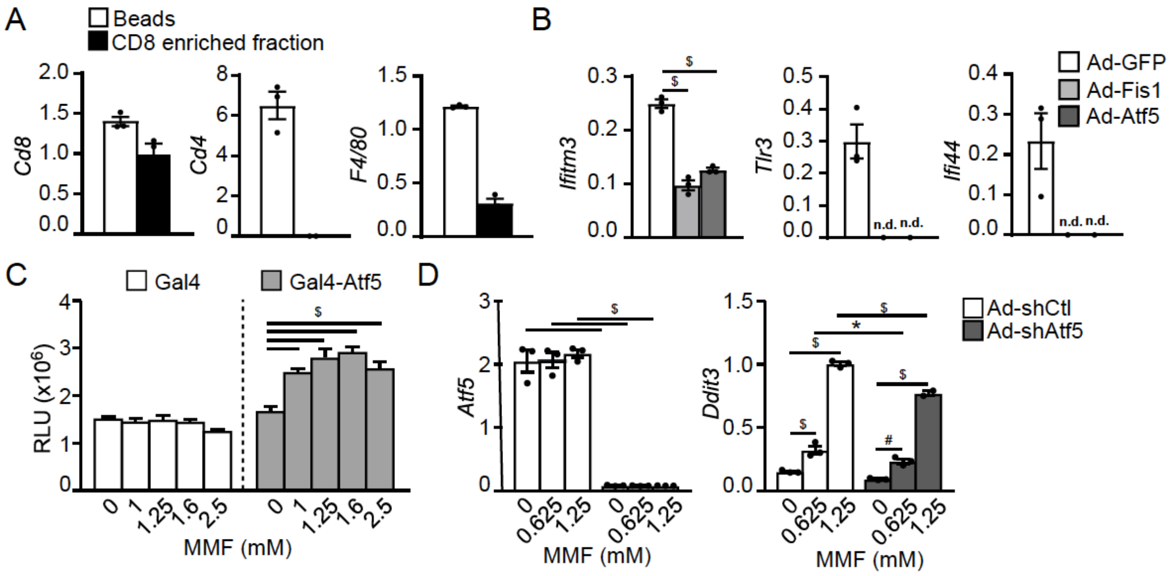

Supplemental Figure 6. Fumarate increases ATF5 activity. (**A**) Relative expression of lymphocyte (*Cd8* and *Cd4*) and macrophage (*F4/80*) markers by real-time PCR. Male mice fed a HFD for 2 months were infected with either Ad-GFP, Ad-Fis1 or Ad-Atf5. Five days after infection, livers were collected, pooled and mechanically dissociation to release hepatocytes and immune cells. $CD8^+$ cells were enriched using a negative selection method that pulled down $CD8^-$ immune cells with magnetic beads. RNA extraction was performed in both the bead and flow-through ($CD8^+$ T cell enriched) fractions to assess the purification efficiency. Experiments conducted in one mouse cohort. **(B)** Relative expression of genes in IFN-I signaling by real-time PCR in $CD8^+$ T cells from livers of Ad-GFP, Ad-Fis1 or Ad-Atf5 infected mice. **(C)** Monomethyl fumarate (MMF) increases the transactivation activity of Atf5. AD293 cells were co-transfected with a luciferase reporter driven by SV40 promoter with 4 copies of the Gal4-binding site and an expression vector for Gal4 control or Gal4-Irf3, together with CMV-β-galactosidase to monitor the transfection efficiency. Cells were treated with MMF at the indicated doses for 16 hours. The luciferase activity was normalized by the β-galactosidase activity to determine relative light unit (RLU). n=5. Statistical analysis: MMF treated vs untreated condition. (**D**) Relative expression of *Atf5* and *Ddit3* by real-time PCR in primary hepatocyte infected with Ad-shCtl or Ad-shAtf5 treated with MMF at indicated doses for 24 hours. n=3. **C**-**D**: experiments repeated 3 times. Values are presented as mean ± SEM. Significance of **A** was determined by unpaired, two-tailed Student's *t* test; **B**-**D** by One-way ANOVA followed by Sidak multiple comparisons test. $^*p<0.05$, $^\#p<0.01$, $^\$p<0.001$.

Supplemental Table 1. Fasting blood chemistry in the Ad-shFis1 vs Ad-shCtl mouse cohort

	Ad-shCtl (n=5)	Ad-shFis1 (n=5)
Body weight (g)	28.60 ± 1.22	28.80 ± 0.70
Triglycerides (mg/dL)	33.12 ± 2.92	32.10 ± 5.82
Free fatty acids (mM)	0.83 ± 0.07	0.83 ± 0.05
Total cholesterol (mg/dL)	130.90 ± 6.17	137.20 ± 5.31
Glucose (mg/dL)	186.20±9.45	192.00±3.96
Insulin (ng/mL)	1.37 ± 0.22	0.83 ± 0.19

C57BL/6J male mice were fed a HFD for 4 weeks starting at 6-week old of age. Data are presented as means ± SEM.

Supplemental Table 2. Fasting blood chemistry in the Ad-Fis1 vs Ad-LacZ mouse cohort

	Ad-LacZ (n=7)	Ad-Fis1 (n=7)
Body weight (g)	34.03 ± 1.00	32.83 ± 0.94
Triglycerides (mg/dL)	78.40 ± 6.13	51.78 ± 9.01*
Free fatty acids (mM)	2.37 ± 0.12	1.70 ± 0.14$^{\#}$
Total cholesterol (mg/dL)	118.80 ± 4.73	103.90 ± 1.95*
Glucose (mg/dL)	128.10±5.53	86.67±4.20$^{\$}$
Insulin (ng/mL)	n.d.	n.d.

C57BL/6J male mice were fed a HFD for 4 weeks starting at 6-week old of age. Data are presented as means ± SEM. Significance was determined by unpaired, two-tailed Student's *t* test. *$p<0.05$, $^{\#}p<0.01$, $^{\$}p<0.001$ for Ad-Fis1 vs Ad-LacZ. n.d.: not determined.

Supplemental Table 3. Fasting blood chemistry in the Ad-Atf5 vs Ad-GFP mouse cohort

	Ad-GFP (n=5)	Ad-Atf5 (n=5)
Body weight (g)	38.58 ± 2.37	37.02 ± 1.97
Triglycerides (mg/dL)	44.73 ± 1.52	34.57 ± 1.63[#]
Free fatty acids (mM)	0.64 ± 0.03	0.60 ± 0.04
Total cholesterol (mg/dL)	196.00 ± 17.90	133.10 ± 11.90*
Glucose (mg/dL)	183.20±7.57	158.30±5.18*
Insulin (ng/mL)	1.53 ± 0.31	0.57 ± 0.16*

C57BL/6J male mice were fed a HFD for 12 weeks starting at 6-week old of age. Data are presented as means ± SEM. Significance was determined by unpaired, two-tailed Student's *t* test. *$p<0.05$, [#]$p<0.01$ for Ad-Atf5 vs Ad-GFP.

Supplemental Table 4. Fasting blood chemistry in the monomethyl fumarate (MMF) treatment mouse cohort

	Vehicle (n=5)	MMF (n=5)
Body weight (g)	36.52 ± 1.25	32.72 ± 1.94
Triglycerides (mg/dL)	56.61 ± 16.58	56.22 ± 3.12
Free fatty acids (mM)	0.72 ± 0.06	0.65 ± 0.01
Total cholesterol (mg/dL)	125.50 ± 11.40	117.00 ± 6.50
Glucose (mg/dL)	187.00±13.48	133.50±8.84*
Insulin (ng/mL)	0.55 ± 0.06	0.40 ± 0.08

C57BL/6J male mice were fed a HFD with or without MMF for 6 weeks starting at 12-week old of age. Data are presented as means ± SEM. Significance was determined by unpaired, two-tailed Student's *t* test. *p<0.05, MMF vs vehicle.

Supplemental Table 5. List of antibodies and reagents

Type	Designation	Vender	Catalog#
Antibody	Rabbit polyclonal anti-Fis1	Proteintech	10956-1-AP
Antibody	Rabbit monoclonal anti-Dmn1l (D8H5)	Cell Signaling	5391
Antibody	Rabbit monoclonal anti-Irf3 (D83B9)	Cell Signaling	4302
Antibody	Rabbit polyclonal anti-FLAG	Sigma-Aldrich	F7425
Antibody	Rabbit monoclonal anti-HA (C29F4)	Cell Signaling	3724
Antibody	Rabbit polyclonal anti-LC3B	Sigma-Aldrich	L7543
Antibody	Rabbit monoclonal anti-Actb (D6A8)	Cell Signaling	8457
Antibody	Rabbit monoclonal anti-Tubb (9F3)	Cell Signaling	2128
Antibody	Rabbit monoclonal anti-SQSTM1/p62 (D6M5X)	Cell Signaling	23214
Antibody	GCN2 antibody	Cell Signaling	3302
Antibody	Rabbit monoclonal Anti-Phospho-GCN2 (Thr899)(E1V9M)	Cell Signling	94668
Antibody	Rabbit monoclonal anti-Lmnb1 (D9V6H)	Cell Signaling	13435
Antibody	Rabbit monoclonal anti-Sdhb (EPR10880)	Abcam	175225
Antibody	Mouse monoclonal anti-CHOP (L63F7)	Cell Signaling	2895
Chemical compound	Paraquat	Sigma-Aldrich	856177
Chemical compound	Valinomycin	Sigma-Aldrich	V0627
Chemical compound	Monomethyl fumarate	Sigma-Aldrich	651419
Chemical compound	cOmplete™ Protease Inhibitor Cocktail	Sigma-Aldrich	1187358000
Chemical compound	MitoSOX Red superoxide indicator	ThermoFisher Scientific	M36008
Chemical compound	MitoReacker Green	ThermoFisher Scientific	M7514
Chemical compound	Seahorse XF24 FluxPak	Agilent	102070-00
Sequence based reagent	Poly I:C (HMW)	Invovogene	Tlrl-pic
Recombinant protein	Recombinant mouse IFNβ1	BioLegend	581302

Assay kit	Infinity^{TM} Triglycerides Liquid Stable Reagent	ThermoFisher Scientific	TR22421
Assay Kit	Cholesterol E	Fujifilm Helthcare	999-02601
Assay Kit	HR Series NEFA-HR(2)	Fujifilm Helthcare	999-34691
Assay Kit	Protein Carbonyl Content Assay kit	Abcam	ab126287
Assay kit	BCA Protein Assay Kit	Pierce	23223, 1859078
Assay kit	Clarity Western ECL	BioRad	170-5061
Assay Kit	Mouse/Rat Insulin Kit	Meso Scale Discovery	K152BZC -1
Assay kit	Bradford Reagent	BioRad	500-0006
Recombinant DNA reagent	pBABE-puro retroviral expression vector (plasmid)	Addgene	1764
Recombinant DNA reagent	IFN-Beta_pGL3 (plasmid)	Addgene	102597
Liquid Medium	Dulcecco's Modified Eagle's Medium, 1g/L glucose	CORNING	10-014-CV
Liquid Medium	Dulcecco's Modified Eagle's Medium, 4.5g/L glucose	CORNING	10-013-CV
Liquid Medium	EBSS	Gibco	14155063
Liquid Medium	Williams' Medium E	Gibco	12551-032
Cell Line	AD293 (human embryonic kidney epithelial cells)	Agilent	240085
Cell Line	Phoenix-AMPHO (human kidney epithelial cells)	ATCC	CRL-3213
Cell Line	Hepa 1-6 (mouse hepatoma cells)	ATCC	CRL-1830
Mouse strain	C57BL6/J	Jackson Laboratory	000664
Mouse diet	Rodent diet 20	PicoLab	5053
Mouse diet	Mouse Diet, High Fat Fat Calories (60%)	Bio-Serv	F3282
Adenovirus	Ad-Atf5	Vector BioLab	#ADV-253209
Adenovirus	Ad-Dmn1l	Vector BioLab	#ADV-257347
Adenovirus	Ad-shCtl: scrambled shRNA control	Vector BioLab	#1122N
Adenovirus	Ad-shFis1	Vector BioLab	shADV-259434
Adenovirus	Ad-shAtf5	Vector BioLab	shADV-253209

Other	Verso cDNA synthesis kit	ThermoFisher Scientific	AB1453B
Other	NucleoSpin RNA Plus kit	Macherey-Nagel	740984
Other	Liberase^{TM} TM	Sigma-Aldrich	5401119001
Other	Percoll	Sigma-Aldrich	17-0891-01
Other	Ficoll Paque Plus	Sigma-Aldrich	GE17-1440-02
Other	TranslT-LT1 Transfection Reagent	Mirus	MIR 2300
Other	Bovine serum albumin	Gemini Bio-Products	700-107P
Other	Mouse CD8T Lymphocyte Enrichment Set	BD	558471
Other	Anti-FLAG M2 affinity Gel	Sigma-Aldrich	A2220

Supplemental Table 6. List of primer sequences used for RT-qPCR

Gene	Forward Sequence	Reverse Sequence
Hspa5	GACTGCTGAGGCGTATTTGG	AGCATCTTTGGTTGCTTGTCG
Irf3	TGCCTCACTCCCAGGAAAAC	GCTTGGCAGTTGTTGAGAAGG
Irf7	CTTCCGAGAACTGGAGGAGTTT	CTTGCCCAAAACCCAGGTAGA
Acox2	CACCCTGACATAGACAGTGAAAG	CTGGGTCACGTTGGATGAGG
Mff	CAGTTGGCAGGCTAAAAAGAGA	GCCCTACGAGTAGAAGACTGG
Fis1	AGGCTCTAAAGTATGTGCGAGG	GGCCTTATCAATCAGGCGTTC
Mfn2	CTTCTCCTCTGTTCCAGTTG	CATCTCGCCAGTTTATATGCAG
Atf4	GCCGGTTTAAGTTGTGTGCT	CTGGATTCGAGGAATGTGCT
Asns	GAGAAACTCTTCCCAGGCTTTG	CAAGCGTTTCTTGATAGCGTTGT
Atf5	TGGGCTGGCTCGTAGACTAT	GTCATCCAATCAGAGAAGCCG
Atf6	CGGTCCACAGACTCGTGTTC	GCTGTCGCCATATAAGGAAAGG
Tom20	GCCCTCTTCATCGGGTACTG	ACCAAGCTGTATCTCTTCAAGGA
Timm8a1	CAGATGACGGAACTTTGCTGG	AAGACGGGTTTGGACTTCTGG
Nbr1	AGCTACCTGGAGATGTTCCGA	AATAGGCATTGAGACTTCTGTGG
Dlat	CTTTAGCCTCCAAAGCGAGAG	AGATTGTAAATGTTCCACCCTGG
Dbt	AGACTGACCTGTGTTCGCTAT	GAGTGACGTGGCTGACTGTA
VPS13c	GAAGCTAAAGTAAAAGCCCACGA	ACACATCAGAGGTGTTGACAATG
Vps13b	CAGTAAAGAGTCTCACGCTACAG	TGTTCCAGGGATGTCACCAGA
Copb1	GCCGAGAACGTGTGCTATAC	GCGAATGATTGTCATCAGGAGTC
Copa	ACCAAATTCGAGACGAAGAGC	CCGATAGTCCCATAACTGGATGA
Sec23a	TCGAGTCGTCTGGAAGCTACA	AGGTGGAAACTGATTCCTTTGG
Snx5	AGGACCGCAGCAAGTTAAGAT	GGGCTCTGAAATGTGGACAGT
Lars2	CATAGAGAGGAATTTGCACCCTG	GCCAGTCCTGCTTCATAGAGTTT
Sars	CGGGTGGATAAAGGAGGGGA	TGCCCGAAATCTGCATCGTC
Rpl22l1	CCTGGAGGTTTCATTTGGACC	ACGTAGCCAATCACGGAGATTA
Rps20	GAGAAGGTTTGTGCGGACTTG	AAATCAATGAGTCGCTTGTGGA
Ddx51	GCTCAAGGGCTCTTTGCTTCA	TGTGCTGATAAGGAGCTGGAT
Dhx34	GAATTGTCCAGAGACTCGTTGT	TGGGAGGTCTTGAGGTGCT
Eer1	TGGACGGACAGAATACACCAT	CTGCATAGTCAAAATAGGTGGCA
Ube2j2	CTTGAATGGCATTATGTTGTCCG	CAGCCTTGTGTTGCACTTAAATC
Dnajb9	TCAGAGCGACAAATCAAAAAGGC	CTATTGGCATCCGAGAGTGTTT
Nars	GAGCTGTATGTATCTGACCGAGA	AAATGGTGGGAAATGGCTCTTT
Vars	GTCCAGCAGTGGGTCAGTTAT	GCAGCAGTAAGGCTGTCAC
Farsb	ATGAAGAGTTTGACGAACTGTGT	GAGGTCGTATCTGTTGGCAGG
Ho1	AAGCCGAGAATGCTGAGTTCA	GCCGTGTAGATATGGTACAAGGA
Ifitm3	CCCCCAAACTACGAAAGAATCA	ACCATCTTCCGATCCCTAGAC
Ifit2	AGTACAACGAGTAAGGAGTCACT	AGGCCAGTATGTTGCACATGG
Ifit1	CTGAGATGTCACTTCACATGGAA	GTGCATCCCCAATGGGTTCT
Aox1	GAGGAAGAATCTCCGACTCACA	TGGTGACTGCTGTACCATGTAG
Mthfd2	AGTGCGAAATGAAGCCGTTG	GACTGGCGGGATTGTCACC
Mthfd1l	GCATGGCCTTACCCTTCAGAT	GTACGAGCTTCCCCAGATTGA
Aldh1l2	ACCAGCCGGGTTTATTTCAAA	ACTCCCACTACTCGGTGGC

Shmt2	TGGCAAGAGATACTACGGAGG	GCAGGTCCAACCCCATGAT
Ifit3	ATGAGTGAGGTCAACCGGGAAT	CATTGTTGCCTTCTCCTCAGAG
Ddx58	AAGAGCCAGAGTGTCAGAATCT	AGCTCCAGTTGGTAATTTCTTGG
Dhx58	CAACACCATCTTGAGCCGGTA	AATTCGCACAAAGCTGTAGGA
Zbp1	TCAGGAAGGCCAAGACATAGCT	TCTGGATGGCGTTTGAATTGG
Usp18	TTGGGCTCCTGAGGAAACC	CGATGTTGTGTAAACCAACCAGA
Ifnb1	CAGCTCCAAGAAAGGACGAAC	GGCAGTGTAACTCTTCTGCAT
Ifna4	TGATGAGCTACTACTGGTCAGC	GATCTCTTAGCACAAGGATGGC
Tlr3	CCTCCAACTGTCTACCAGTTCC	GCCTGGCATAGTTATTGTGC
Cd4	TGCCCCCACCGGATGCAGAA	CAGGTGGTGGGCTGCAGGTG
Cd8	TGCAAATGTCCCAGGCCGCT	TGTAGCTTCCTGGCGGTGCC
F4/80	CTTTGGCTATGGGCTTCCAGTC	GCAAGGAGGACAGAGTTTATCGTG
Ifi44	CCTGGTTCAGCAAACACGAGT	TGGCCTTGATGGAATATGTCCT
36b4	AGATGCAGCAGATCCGCAT	GTTCTTGCCCATCAGCACC

Supplemental Table 7. Description of animal cohorts

Cohort info	Cohorts/ repeats*	Age	Gender	Animal	Figures
NC & 8W-HFD ZT2 & ZT14	2	14-week-old	Male	4/group	1B-1D, S1A
NC & 12W-HFD	2	18-week-old	Male	5/group	S1B, S1C
24 hours fasted or 19 hours fasted/5 hours refed	2	8-week-old	Male	3/group	1A
4W-HFD Ad-Lacz or Ad-Fis1 infected	2	9-week-old	Male	7/group	2A-2E, S3H
12W-HFD Ad-GFP or Ad-Fis1 infected	1	18-week-old	Male	6/group	2F-2H, 4C
4W-HFD Ad-shCtl or Ad-shFis1 infected	2	9-week-old	Male	5/group	S2A, S2B, S2D, S2E, S3I
11W-HFD Ad-GFP or Ad-Atf5 infected	2	17-week-old	Male	6/group	6A-6E
8W-HFD Ad-GFP or Ad-Fis1 or Ad-Atf5 infected	2	15-week-old	Male	2/group	S6A, S6B
6-week-HFD, 45mg MMF per Kg body weight	2	18-week-old	Male	5/group	6F

*For experiments performed in two cohorts, data from one of the two were presented. Similar results were observed between two experiments. NC: normal chow; HFD: high fat diet.

Supplemental Dataset 1 (separated file). List of differentially expressed genes (FDR<0.001) identified by RNA-seq comparing Ad-Fis1 vs Ad-LacZ infected primary hepatocytes.

Supplemental Dataset 2 (separated file). List of differentially accumulated metabolites ($p<0.05$) comparing Ad-Fis1 vs Ad-LacZ infected primary hepatocytes.

Supplemental Dataset 3 (separate file). List of transcription factors identified by Homer analysis of up- or down-regulated genes (Ad-Fis1 vs Ad-LacZ) identified by RNA-seq.
